# Management of transition dipoles in organic hole-transporting materials under solar irradiation for perovskite solar cells

**DOI:** 10.1038/s41467-018-06998-1

**Published:** 2018-10-31

**Authors:** Song Ah Ok, Bonghyun Jo, Sivaraman Somasundaram, Hwi Je Woo, Dae Woon Lee, Zijia Li, Bong-Gi Kim, Jong H. Kim, Young Jae Song, Tae Kyu Ahn, Sanghyuk Park, Hui Joon Park

**Affiliations:** 1 Department of Energy Systems Research, Ajou University, Suwon, 16499 Republic of Korea; 2 Department of Energy Science, Sungkyunkwan University, Suwon, 16419 Republic of Korea; 3 Department of Chemistry, Kongju National University, Kongju, 32588 Republic of Korea; 4 SKKU Advanced Institute of Nanotechnology (SAINT), Sungkyunkwan University, Suwon, 16419 Republic of Korea; 5 Department of Molecular Science and Technology, Ajou University, Suwon, 16499 Republic of Korea; 6 Department of Organic and Nano System Engineering, Konkuk University, Seoul, 05029 Republic of Korea; 7 Center for Integrated Nanostructure Physics, Institute for Basic Science (IBS), Sungkyunkwan University, Suwon, 16419 Republic of Korea; 8 Department of Physics, Sungkyunkwan University, Suwon, 16419 Republic of Korea; 9 Department of Nano Engineering, Sungkyunkwan University, Suwon, 16419 Republic of Korea; 10 Department of Electrical and Computer Engineering, Ajou University, Suwon, 16499 Republic of Korea

## Abstract

In organic hole-transporting material (HTM)-based *p*−*i*−*n* planar perovskite solar cells, which have simple and low-temperature processibility feasible to flexible devices, the incident light has to pass through the HTM before reaching the perovskite layer. Therefore, photo-excited state of organic HTM could become important during the solar cell operation, but this feature has not usually been considered for the HTM design. Here, we prove that enhancing their property at their photo-excited states, especially their transition dipole moments, can be a methodology to develop high efficiency *p−i−n* perovskite solar cells. The organic HTMs are designed to have high transition dipole moments at the excited states and simultaneously to preserve those property during the solar cell operation by their extended lifetimes through the excited-state intramolecular proton transfer process, consequently reducing the charge recombination and improving extraction properties of devices. Their UV-filtering ability is also beneficial to enhance the photostability of devices.

## Abstract (web summary)

In perovskite solar cells, the excited state property of hole-transport layer is not usually considered for the devices. Here the authors design organic hole-transport materials with high transition dipoles having extended lifetime at the excited states to improve the charge extraction of the devices.

## Introduction

Photovoltaic (PV) devices utilizing organometal trihalide perovskite materials as light absorbers have been highlighted as emerging PVs^1^. Since the first demonstration of solid-state perovskite solar cells (PSCs) in 2012 ^2,3^, of which the liquid electrolyte of perovskite-sensitized mesoporous(mp)-structure-based electrochemical cell^4^ was successfully replaced to the solid hole-transporting-material (HTM), the unceasing optimization of each layer of PSC so far has led their power conversion efficiency (PCE) to surpass 22% now^1,5^. Although those mp-structured architectures have taken a leading role in raising their efficiency, the necessity for the laborious high-temperature annealing process to prepare an efficient mp-structure has aroused intensive researches for the alternative architectures without the mp-structure, so-called planar configurations having simple and low-temperature processibility feasible to the low-cost flexible PSCs.

The planar configuration PSC devices can be constructed to be either an *n−i−p* type or *p−i−n* type, in which a perovskite photoactive layer is directly formed on flat electron-transporting interlayer (ETL) or hole-transporting interlayer (HTL), respectively, prepared on a transparent conducting oxide electrode. Among those, *n−i−p-*type planar configuration PSCs, which utilize TiO_2_, SnO_2_, or ZnO as an ETL, often exhibit hysteretic behaviors showing different current density−voltage (*J*−*V*) characteristics depending on the scan rate and direction, which are believed to be related to various factors such as trap states, unbalanced charge transport, and the charge accumulation induced by ion migration in the perovskite layer^6–8^. On the contrary, it was shown that those hysteretic behaviors could be mitigated by casting fullerene or its derivative on top of the perovskite layer as an ETL of *p−i−n* planar PSCs, because fullerene and its derivative could passivate defect sites and grain boundaries of perovskite layer^9^.

Since the first report of *p−i–n* planar configuration PSC having 3.9% PCE in 2013 ^10^, in which perovskite was sandwiched between poly(3,4-ethylenedioxythiophene):poly(styrenesulfonate) (PEDOT:PSS) HTL and fullerene derivative ETL, their performances have been significantly improved to around 20% nowadays with the continuous endeavor to replace the PEDOT:PSS, having long-term stability issue due to its acidic nature, to better performing new types of HTLs. In this context, various inorganic semiconductors having p-type characteristics such as NiO^11–14^, CuI^15^, Cu_2_O^16–18^, CuSCN^19,20^, PbS^21^, and graphene oxide^22^ have shown great promise to efficiency and stability, but the organic semiconductor-based HTMs, having outstanding processibility for the simple one-step solution-process at low temperatures, also deserve further studies.

Two types of organic semiconductor-based HTMs, small-molecule type (e.g. spiro-OMeTAD derivatives^23^ and triarylamine derivatives^24^) and polymer type (e.g. PTAA^25,26^, poly-TPD^27^, PCDTBT^28^, and thiophene derivative^29^), have been applied to *p−i−n* PSCs, and they could be utilized alone^23–26,29^ or additionally inserted between inorganic semiconductor HTL and perovskite active layer^27,28^. Even though various types of organic HTMs have been suggested as mentioned, for designing efficient organic HTMs of *p−i−n* PSCs, in common, well-aligned highest occupied molecular orbital (HOMO) energy levels to the valence band edge of perovskite for efficient hole extraction, relatively high hole mobility for facilitating hole-transport, and the superior film quality of both HTL and perovskite after casting perovskite solution on those HTLs are required.

Especially in this architecture, the incident light has to pass through the HTLs before reaching the photoactive layer, and thus their bandgap should be high enough to have their absorption in UV region for maximizing the transmitted light to the perovskite in visible and near-IR region of the solar spectrum. This also means that, under the solar illumination condition, fairly large amount of light energy, at least higher than their bandgap (e.g. UV region), would be continuously applied to HTLs, and therefore photo-excited states of organic HTMs, which have been usually overlooked for HTM design until now, could become important during the solar cell operation.

One of the noticeable changes that we can observe from the energy-state difference in organic semiconductors is the alteration of electron distribution within the molecules, which could induce the variation of molecular dipole moment (e.g. transition dipole)^30,31^. Until now, various interlayers that can induce dipoles have been often introduced into the PSCs to increase their built-in potential, subsequently enhancing the electric field for the separation of photo-generated excitons and the facilitation of charge extraction^32^; however, the effect of intrinsic dipole states of organic HTMs in *p−i−n* planar PSCs under the solar illumination condition has never been considered.

In this work, we systematically investigate and establish a molecular design principle to utilize the transition dipole moments of HTMs at photo-excitation for the performance enhancement of *p−i−n* configuration PSCs. For this purpose, the molecules, which not only have the aforementioned basic properties of HTMs but also can maximize the transition dipole moment effect under the solar spectrum by their structural properties that follow the excited-state intramolecular proton transfer (ESIPT) process^33–35^, are synthesized. The ESIPT process accompanies transient chemical variations and therefore the electronic property changes, particularly the dipole moment and polarizability^36,37^. Applying an electron-donating or accepting moiety to ESIPT-molecule, thus, can be a promising molecular design approach to enhance their transient alternations^33,38,39^. Finally, we can investigate the merits of transition dipole moment to the performances of PSCs. We believe that our suggestion can be an effective strategy to develop efficient HTMs for high-performance PSCs.

## Results

### Design of organic small-molecule HTMs

**Fig. 1:**
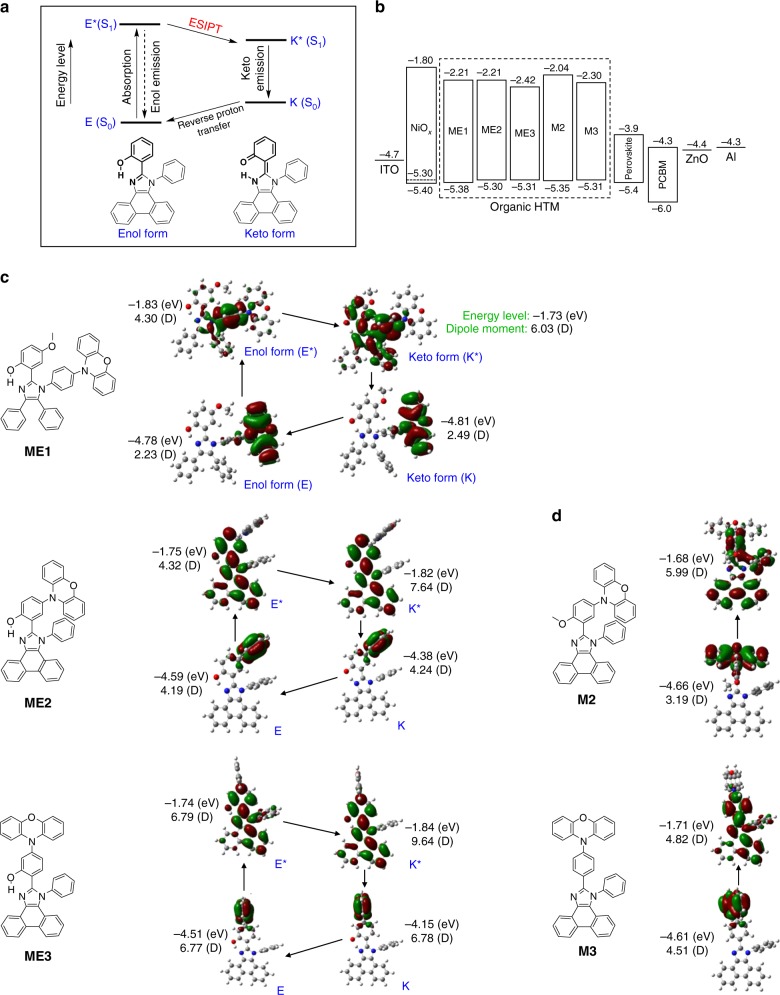
Molecular characteristics of organic HTMs. **a** Schematic representation of four-level photocycle of the ESIPT process. **b** Energy levels of organic HTMs measured by CV and absorbance; energy levels of the other layers composing perovskite solar cell are also added^32^. **c** Chemical structures of the designed ESIPT-active HTMs (ME1, ME2, and ME3) and their orbital diagrams in enol and keto forms calculated by DFT. **d** Chemical structures of the designed non-ESIPT HTMs (M2 and M3) and their orbital diagrams calculated by DFT. M2 and M3 are prepared as non-proton-transfer model compounds of ME2 and ME3. Beside all orbital diagrams, theoretically calculated energy levels and dipole moments are added

To investigate the effect of transition dipole moment of organic HTM at the photo-excitation on the performances of PSC, we have designed and synthesized five phenoxazine- and imidazole-containing organic small-molecule semiconductors (ME1, ME2, ME3, M2, and M3) (Fig. 1c, d), having a different degree of dipole moment, and those of conventional HTMs such as spiro-OMeTAD and PTAA were also considered. The synthetic routes for making those five molecules are shown in Supplementary Fig. 1 and 2. In brief, after the one-pot imidazole synthesis using Debus−Radziszewski method with bromosalicylaldehyde, benzil, aniline, and ammonium acetate in acetic acid, the phenoxazine coupling reaction was followed in high reaction yield (64%). The detailed synthetic information and material characterization can be found in the Methods section and the Supplementary Information; ^1^H NMR, ^13^C NMR, mass, differential scanning calorimetry, and thermogravimetric analysis (TGA) are in Supplementary Figs. 3−18 and Supplementary Notes 1−5. It is noteworthy that all the molecules could be obtained using a simple two-step method from readily available commercial starting materials, in contrast to the complex and expensive procedures of conventional organic small-molecule HTM such as spiro-OMeTAD^23^.

In the design of the HTMs, phenoxazine was selected because electron-rich phenoxazine derivatives exhibit high hole mobility and conductivity, which qualify them as proper candidates for the efficient HTMs of PSCs^40,41^. In addition, to magnify the hole-transporting property of the phenoxazine moiety and to optimize energy levels of the HTMs for visible transparency and energy-level alignment to perovskite, tetraphenylimidazole (e.g. for ME1) or phenanthroimidazole (e.g. for ME2, ME3, M2, and M3) structure was introduced since the imidazole derivatives not only act as charge-transporting materials as a result of their ambipolar characteristic, but also form an electron donor−acceptor configuration with phenoxazine due to their relative electron-deficiency^42–44^. The hole mobility of the designed HTMs was evaluated by a space-charge limited current method using the Mott–Gurney law: *J* = 9*ε*_o_*ε*_r_*μV*^2^/8*L*^3^, where *ε*_o_*ε*_r_ is the permittivity of the component, *μ* is the carrier mobility, and *L* is the thickness^24^, and they have comparable hole mobility with the conventional HTM such as spiro-OMeTAD (Supplementary Fig. 19). Additionally, those molecules were designed more asymmetric than the conventional HTMs such as spiro-OMeTAD and PTAA to have higher dipole moments. More importantly, to design the molecules preserving high transition dipole moment effect under the solar spectrum, those were further manipulated by the introduction of intramolecular hydrogen (H) bond, which could modulate their molecular structures between enol (E) and keto (K) form through a process called ESIPT^33–35^.

ESIPT is a phototautomerization process that occurs in the excited state of intramolecularly H-bonded molecules (Fig. 1a)^45,46^. The molecules following ESIPT process are generally more stable in E forms in the ground states (*S*_0_) but in K* forms in the excited states (*S*_1_), and thus their photoexcitation is instantly accompanied by the four-level cyclic proton-transfer reactions (E → E* → K* → K → E) enabled by the intramolecular H-bonds^47,48^. The existence of the stable excited state is an important feature of ESIPT material, because the inherent four-level character of ESIPT provides the straightforward population inversion in K* state (Fig. 1a). Consequently, the effective lifetime of photo-excited state could be magnified in ESIPT molecule as K* form, compared to the conventional organic semiconductors (i.e. this will be further confirmed with time-resolved photoluminescence (TRPL) results of molecules, later), and the molecular property in K* form, predominantly existing at the excited state, could become crucial to the operation of *p−i−n* PSC, applying ESIPT material as an HTM, under broadband solar irradiation. ME1, ME2, and ME3 were prepared to have high transition dipole moment in the ESIPT process-driven K* form, and M2 and M3 were synthesized as non-proton-transfer model compounds of ME2 and ME3 for comparison, of which hydroxyl group was replaced with methoxy group and just hydrogen, respectively.

Especially, ME1, ME2, and ME3 were designed to have a different level of dipole moment by controlling their degree of conjugation, and this was possible by changing the position of electron-rich functional group, phenoxazine, affecting the planarity of the molecule. From ME1 to ME3, the angle between a backbone of central imidazole and a phenoxazine unit is getting larger (e.g. angle between imidazole and phenoxazine: ME1 < ME2 < ME3), which increases the electron confinement effect within the molecule, consequently providing higher dipole moment. Moreover, phenyl rings on the bottom of the imidazole were crosslinked in ME2 and ME3, and therefore they have a full-flat conformation around the central imidazole ring, further increasing their effective conjugation length and dipole. In contrast, ME1 has the twisted conformation decreasing its dipole moment due to the flexible phenyl groups within the molecule. As depicted in Supplementary Fig. 1, two phenyl rings at the 4- and 5-position of the central imidazole can be easily crosslinked to each other by changing the starting material (*α,β*-diketone) from benzil to 9,10-phenanthrenequinone.

### DFT calculation of organic molecules

**Fig. 2:**
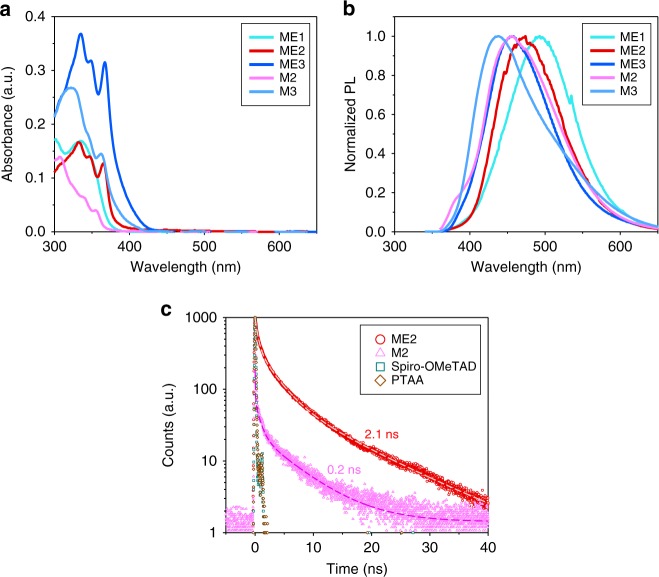
Photophysical properties of organic HTMs. **a** Absorbance and **b** steady-state photoluminescence of designed organic molecules in thin films casted on quartz (cyan, red, blue, pink, and sky-blue lines are for ME1, ME2, ME3, M2, and M3, respectively). **c** Time-resolved photoluminescence of organic molecules casted on quartz (red circle, pink triangle, green square, and brown diamond symbols are for ME2, M2, spiro-OMeTAD, and PTAA, respectively)

The dipole moments of the designed molecules including their energy levels and frontier orbital distributions were calculated using the density functional theory (DFT) method with the Gaussian 09 program at the B3LYP (Becke’s three-parameter exchange function and Lee−Yang−Parr correlation function) level with 6-31G(d, p)^49,50^ basis sets. Figure 1c, d shows the HOMO and the lowest unoccupied molecular orbital (LUMO) levels of the synthesized molecules. The calculated energy-level values of the molecules are added to Fig. 1c, d, and summarized in Supplementary Fig. 20b. Their overall trends are well-matched to the experimentally determined energy levels (Fig. 1b and Supplementary Fig. 20a) by cyclic voltammetry (CV) (Supplementary Fig. 21) and the absorbance (Fig. 2a). The orbital distributions and energy levels of spiro-OMeTAD and PTAA (theoretically and experimentally) are in Supplementary Fig. 22. The attained energy levels confirm that all those synthesized molecules have adequate potential to work as HTMs in PSCs efficiently, because their HOMO levels comparable to the valance band edge of perovskite (−5.4 eV) are advantageous to extract hole from the perovskite layer and their small LUMO levels effectually prevent electron transport from the perovskite to the HTMs.

The calculated dipole moments of designed materials are added to Fig. 1c, d, and summarized in Table 1. Additionally, the dipole moments of conventional HTMs (spiro-OMeTAD and PTAA) were also estimated by DFT calculation and added to Supplementary Fig. 22e, 22f, and Table 1. In all synthesized molecules, the electron densities in HOMOs are mainly localized in the phenoxazine region with some extending to the adjacent aromatic rings, while those in LUMO spread over the central tetraphenylimidazole (e.g. for ME1) or phenanthroimidazole (e.g. for ME2, ME3, M2, and M3) core. Similar to the previous DFT studies on imidazole-, benzoxazole-, and benzoylmethyleneindol-based ESIPT molecules^51–53^, the calculated dipole moments increase on excitation, and the dipole moments in K* (and K) forms show higher values than those in E* (and E) forms. Consequently, in all ESIPT-active molecules (ME1, ME2, and ME3), the dipole moments at K* excited states, expected to be important under the solar illumination due to their increased effective lifetimes, are higher than those at other states, and ME3 and ME1 molecules have the highest and lowest K* dipole moments, respectively, according to the design principles explained earlier [dipole moment at K*(*S*_1_): ME3 (9.64 D) > ME2 (7.64 D) > ME1 (6.03 D)]. M2 and M3 molecules that do not have the ESIPT phototautomerization as non-proton-transfer model compounds of ME2 and ME3 have generally lower dipole moments, but those are still higher than the dipole moments of conventional HTMs (spiro-OMeTAD and PTAA) due to their asymmetric molecular designs. The difference of transition dipole moment among the molecules at excitation was experimentally confirmed by fluorescence anisotropy, and this will be discussed in the following section.

**Table 1:** Dipole moments for ME1, ME2, ME3, M2, M3, spiro-OMeTAD, and PTAA

Molecule	*μ*_g_ (E)^a^	*μ*_e_ (E*)^b^	*μ*_g_ (K)^c^	*μ*_e_ (K*)^d^	Δ*μ*_ge_^e^	*r* ^f^
ME1	2.23	4.30			2.07	0.047
			2.49	6.03	3.54	
ME2	4.19	4.32			0.13	0.061
			4.24	7.64	3.40	
ME3	6.77	6.79			0.02	0.091
			6.78	9.64	2.86	
M2	3.19^g^	5.99^g^			2.80	0.023
M3	4.51^g^	4.82^g^			0.31	0.026
Spiro-OMeTAD	0.28^g^	0.72^g^			0.44	0.005
PTAA	0.27^g^	1.98^g^			1.71	0.002

^a^Enol form ground-state dipole moment (theoretical value by DFT, unit: D)

^b^Enol form excited-state dipole moment (theoretical value by DFT, unit: D)

^c^Keto form ground-state dipole moment (theoretical value by DFT, unit: D)

^d^Keto form excited-state dipole moment (theoretical value by DFT, unit: D)

^e^Difference between the ground- and excited-state dipole moment (theoretical value by DFT)

^f^Fluorescence anisotropy (experimental value)

^g^Because M2, M3, spiro-OMeTAD, and PTAA are not ESIPT-molecules, they have only one ground and excited states of their own molecular structures. Their dipole moment values are added here for convenience

### Photophysical properties of organic thin films

The photophysical properties of the synthesized molecules were characterized by UV−Vis absorption and PL measurement in film (Fig. 2a, b) and solution (Supplementary Fig. 23) states at room temperature. As shown in Fig. 2a, the prepared molecules showed intense absorption mainly in the UV region because of the limited conjugation length in each molecule, which was deliberately designed to be transparent in visible region. However, as the effective conjugation lengths of ESIPT molecules are enlarged to increase their dipole moments (e.g. ME1 → ME2 → ME3), their absorption efficiencies increase, and moreover their absorption bands are red-shifted into the visible region, which is expected to reduce the transmitted light into the perovskite layer in *p−i−n-*type PSCs. Absorbance spectra of spiro-OMeTAD and PTAA are in Supplementary Fig. 22c.

Meanwhile, upon *S*_0_ → *S*_1_ (ππ*) excitation (*λ*_max_ = 330 nm) of ME1, ME2, and ME3 molecules, intense emissions at 455, 470, and 490 nm and shoulder-like very weak emissions at shorter wavelengths were observed (Fig. 2b and Supplementary Fig. 24). The excitation spectra for both emissions, surveyed at their respective bands, are equal as the lowest absorption band, suggesting that both fluorescence bands come from the same ground-state species^34,35^. Thus, we can distinctly ascribe the shoulder-like bands to the normal E* fluorescence and the bands at 455, 470 and 490 nm to the K*-tautomer fluorescence^54^, and confirm that the K^*^ forms are predominantly present in the excited state of each molecule. In comparison, M2 and M3, non-proton-transfer model compounds of ME2 and ME3, respectively, which lack a hydroxyl proton, show only a normal Stokes-shifted fluorescence at around 455 and 435 nm.

From the TRPL signals of organic molecules (casted on quartz), we could confirm that the effective lifetime of photo-excited state of ESIPT molecule significantly increased. As representatives, PL decays of ME2 and its non-proton-transfer model compound M2, characterized by a time-correlated single photon counting (TCSPC) system, are shown in Fig. 2c, and those of other conventional organic HTMs (spiro-OMeTAD and PTAA) are also considered. The decay curve of each sample was convoluted using exponential functions and the results are in Supplementary Table 1. As shown, the calculated PL lifetime (*τ*_ave_) of ME2 (2.1×10^−9^ s) was obviously longer than those of other organic HTMs (M2: 0.2×10^−9^ s, spiro-OMeTAD and PTAA: too small to fit curves), which demonstrated that effective lifetime of photo-excited state could be maximized through the ESIPT process. Consequently, the extended lifetime of the photo-excited state of our ESIPT molecule (from TRPL), along with the predominant population of K* form at the excited state (from steady-state PL and absorbance), supports that the property in K* form, especially the enhanced transition dipole moment in this work, could become important to the operation of *p−i−n* PSC, which applies the designed ESIPT-active molecule as a HTM, under solar illumination including UV light.

**Fig. 3:**
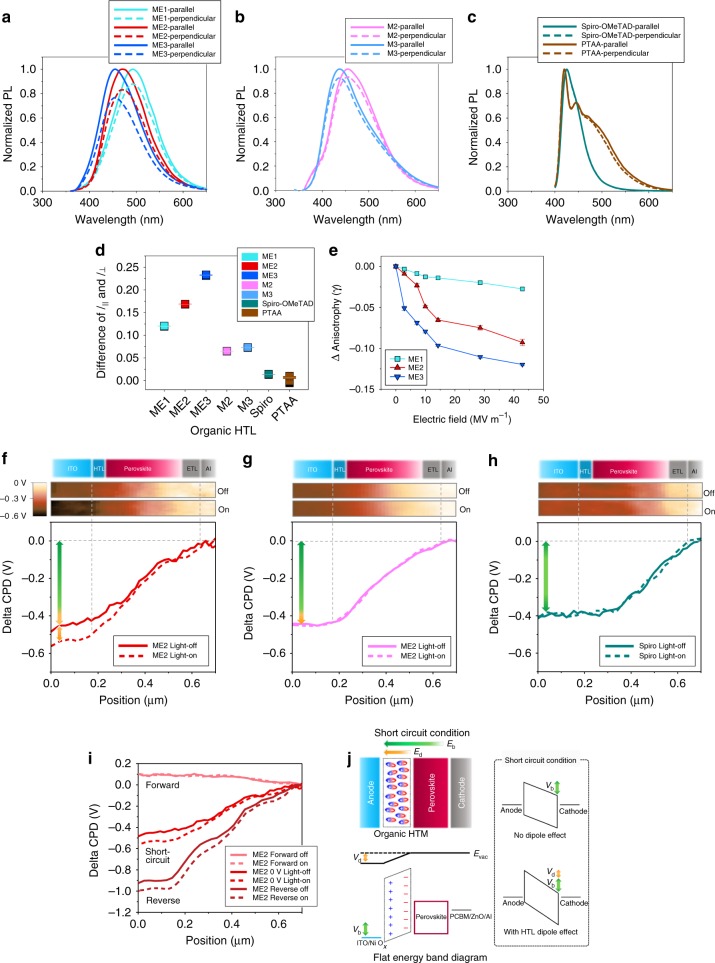
Characterization of transition dipoles in organic thin films. **a**–**e** Fluorescence anisotropy of organic HTM thin films: **a** ESIPT-active HTMs (ME1, ME2, and ME3). **b** Non-ESIPT-active HTMs (M2 and M3). **c** Spiro-OMeTAD and PTAA. **d** PL intensity difference between *I*_║_ and *I*_⊥_ around maximum point (±5 nm range). **e** PL anisotropy decreases of organic HTM thin films depending on the applied electric field. **f–i** Potential profiles of the devices with and without light illumination measured by KPFM (contact potential difference, i.e. CPD, between the tip and the cross-sectional surface of the device). CPD maps are added: **f** Device with ME2 at short-circuit condition. **g** Device with M2 at short-circuit condition. **h** Device with spiro-OMeTAD at short-circuit condition. **i** Device with ME2 at various bias conditions (forward: around 0.8 V and reverse: around −0.5 V). **j** Energy-level diagrams of the device about the dipole effect of organic HTM (*V*_b_ and *E*_b_: built-in potential and electric field, *V*_d_ and *E*_d_: improved built-in potential and electric field by the dipole effect of HTM)

The transition dipole moments of molecules in thin film at photo-excitation could be evaluated by measuring their fluorescence anisotropy^55^, and those results are represented in Fig. 3a–d and summarized in Table 1. The anisotropy (*r*) was obtained by the following equation: *r* = (*I*_║_ − *I*_⊥_)/(*I*_║_ + 2*GI*_⊥_), where *I*_║_ (defined as parallel) and *I*_⊥_ (defined as perpendicular) are PL intensities with polarizers of parallel and perpendicular geometries with respect to the excitation light source, respectively. In our experiments, *G* value was calibrated to be 1.4. The measured anisotropy values show the excellent correlation with the calculated transition dipole moments by DFT. ME3 and ME1 showed the highest and lowest values among the ESIPT-active molecules (ME3: 0.091 > ME2: 0.061 > ME1: 0.047), and the non-proton-transfer model compounds, M3 (0.026) and M2 (0.023), had relatively lower anisotropy than the ESIPT-active molecules. Moreover, the conventional HTMs, spiro-OMeTAD (0.005) and PTAA (0.002), showed negligible anisotropy. For a reference, the differences between *I*_║_ and *I*_⊥_ (at the wavelength range within ±5 nm showing the maximum intensity) are plotted in Fig. 3d.

### Transition dipole moment of molecules under electric field

Because we investigate the effect of dipole moment of molecules in tens of nanometer thick HTLs, different to the conventional ultra-thin dipole layers^32^, and the solar cells are operated under bias and light, the variation of dipole moment effect within the HTM thin film under electric field and light illumination needs to be confirmed first. The HTM thin films in nonionic homogeneous solid state are expected to have induced dipole moment aligned with the electric field (defined by the multiplication of polarizability and electric field), mainly from the electronic polarization in the molecules, fast enough due to the displacement of electron clouds (approximately 10^−15^ s)^56^, with partial contribution of the vibrational polarization, rather than the orientation polarization, slow and restricted by the lattice forces in solid^56–61^. Especially, the asymmetric donor−acceptor type organic conjugated molecules like the designed HTMs, which are intrinsically polar (i.e. having high permanent dipole moment), are known to have high electronic polarizability due to the presence of delocalized *π*-electrons and their high polarity^57–59^.

**Fig. 4:**
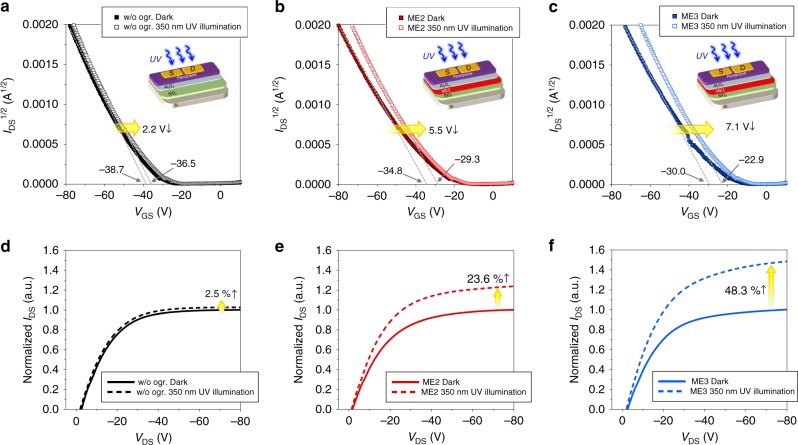
OFETs utilizing organic HTMs as gate dielectric under UV illumination. **a**–**c** Transfer characteristics (*I*_DS_ vs. *V*_GS_) at *V*_DS_ = −80 V for devices **a** without organic HTM, **b** with ME2, and **c** with ME3. All transfer characteristics are about off-to-on scan direction. Data obtained by on-to-off scan direction are in Supplementary Fig. 25. **d**–**f** Output characteristics (*I*_DS_ vs. *V*_DS_) at *V*_GS_ = −75 V for devices **d** without organic HTM, **e** with ME2, and **f** with ME3

The induced dipole moment of organic polar molecules in tens of nanometer scale thin films aligned with the electric field could be proven by the performance improvement of organic field-effect transistors (OFETs), of which those films were inserted to gate dielectric^62–64^. The additional charge accumulation at the interface between the semiconductor and dielectric, induced by the aligned dipoles of organic polar molecules in the dielectric layer under the gate voltage, decreased threshold voltage (*V*_T_) and increased drain current (*I*_DS_) of OFETs^62–64^. Similar to those approaches, OFETs using pentacene and SiO_2_/ESIPT-active ME2 (or ME3)/Al_2_O_3_ as semiconductor and dielectric layer, respectively, were fabricated, and, additionally, UV light (350 nm, 100 μW cm^−2^) was exposed to confirm the effect of the transition dipole moment at the excitation under electric field (Fig. 4a–f). Al_2_O_3_ layer was added to exclude the potential trapping site effect at the interface between the semiconductor and dielectric, inducing a hysteresis^62–64^. Figure 4a, b shows the transfer characteristics of OFETs with and without ME2 layer, and 3.9 V reduction of *V*_T_ (from −38.7 to −34.8 V) was observed by the induced dipole moment in ME2 thin film under the vertical electric field of gate voltage. This reduction was more pronounced in the OFET with ME3 having the highest dipole moment, and *V*_T_ was further reduced to −30.0 V (Fig. 4c). This result represents that ME2 and ME3 thin films can be polarized to have induced dipole moment aligned to the electric field. More importantly, at the light illumination, ME2- and ME3-based OFETs showed the remarkable additional reduction of *V*_T_ from −34.8 to −29.3 V (5.5 V reduction) and from −30.0 to −22.9 V (7.1 V reduction), respectively, compared to that of OFET without the organic dielectric (2.2 V reduction, from −38.7 to −36.5 V), and they also showed much larger saturation *I*_DS_ increases (ME2: 23.6% in Fig. 4e, ME3: 48.3% in Fig. 4f), compared to that of OFET without the organic dielectric (2.5% increase in Fig. 4d). These photo-enhancement effects clearly demonstrate that, under light illumination, the transition dipole moments of ME2 and ME3 at excitation, having higher values than their ground-state dipole moments, are more effective to provide stronger induced dipole moments aligned with the electric field for the additional charge accumulation at the interface. For reference, *V*_T_ reduction and *I*_DS_ increase from the OFET without organic dielectric under illumination is attributed to the photoconductivity effect. Additionally, the OFETs fabricated with Al_2_O_3_ layer did not show noticeable hysteresis in their transfer characteristics, scanned in both off-to-on and on-to-off directions (Supplementary Fig. 25a−f), but those without Al_2_O_3_ layer exhibited pronounced hysteresis (Supplementary Fig. 25g and 25h), as expected.

The alignment of the transition dipole moment at excitation by the applied electric field can be further confirmed by the PL anisotropy measurement. It is well known that the PL anisotropy of the sample contains the dipole orientation of the sample^55,65^. Especially chromophores in thin film have little reabsorption and zero probability of rotation. Therefore, it will reflect the relaxation by the dipole arrangement of the excitons. We have estimated the anisotropy of the films (ME1, ME2, and ME3) with the increase of applied electric field at the corresponding PL maxima (Fig. 3e). As we increased the applied electric field of the film, we observed the gradual decrease of the anisotropy, and the anisotropy change of the ME3 film was the largest. The lower anisotropy value at high electric field compared to that at zero electric field indicates the fast rearrangement of transition dipole. Therefore, we conclude that the steady-state PL loses the anisotropy as the applied electric field increases due to the strong dipole rearrangements of ME3 film.

### Performances of PSCs with organic HTMs

To explore the effect of transition dipole moments of HTMs on the performances of *p−i−n* configuration PSCs, the devices with the following structure, ITO/NiO_*x*_/organic HTM/perovskite/PCBM/ZnO/Al, were prepared by applying various organic HTMs having different levels of dipole moments (ME1, ME2, ME3, M2, M3, spiro-OMeTAD, and PTAA). In this configuration, thin NiO_*x*_ layer (around 20 nm) was commonly added to the devices, underneath the organic HTMs, due to its merits improving open-circuit voltage (*V*_oc_) and stability of devices^11^. The properties of perovskite layers prepared on organic HTMs did not show any noticeable differences from those without the organic HTMs, as confirmed by scanning electron microscopy (SEM), X-ray diffraction, UV−Vis absorbance and PL (Supplementary Fig. 26). The cross-sectional SEM image of PSC device is shown in Supplementary Fig. 27.

As explained in the former section, the organic polar molecule thin film can have induced dipole moment aligning with the electric field, and thus, at short-circuit condition, the organic HTL has the dipole moment directed toward the ITO anode-side due to built-in electric field between electrodes, which results in an increase in the work function of anode (Fig. 3j). To confirm the work function variation of anode originated from the dipoles, the cross-sectional potential profiles of the devices were characterized by Kelvin probe force microscope (KPFM), and the measured contact potential difference (CPD) between the tip of the cantilever and the cross-section surface of the device utilizing ME2, M2 or spiro-OMeTAD as an HTL is shown in Fig. 3f–h, as representatives (CPD is the difference of work functions between the tip and the surface), where zeros are set to the Al electrode for comparison. To rule out sample degradation effects that could be originated during the measurement and cleaving process, *J*−*V* characteristics of devices were examined before (pristine device) and after (cleaved device) KPFM measurement, and those of one of the devices is shown in Supplementary Fig. 28, representing no significant degradation effects. All KPFM results show that CPD profiles monotonically decrease, representing homogeneous electric field through the devices, consistent with the literature^66^. KPFM results clearly show that the built-in potential of the device with ME2, having the strong transition dipole moment as well as the extended lifetime of transition dipole by the ESIPT process, increases at light illumination condition; however, those with M2 and spiro-OMeTAD are not noticeably changed depending on the light illumination. In addition, it was also confirmed that the built-in potential variation among the devices was well-matched with the degree of dipole of HTMs (ME2 > M2 > spiro-OMeTAD).

**Fig. 5:**
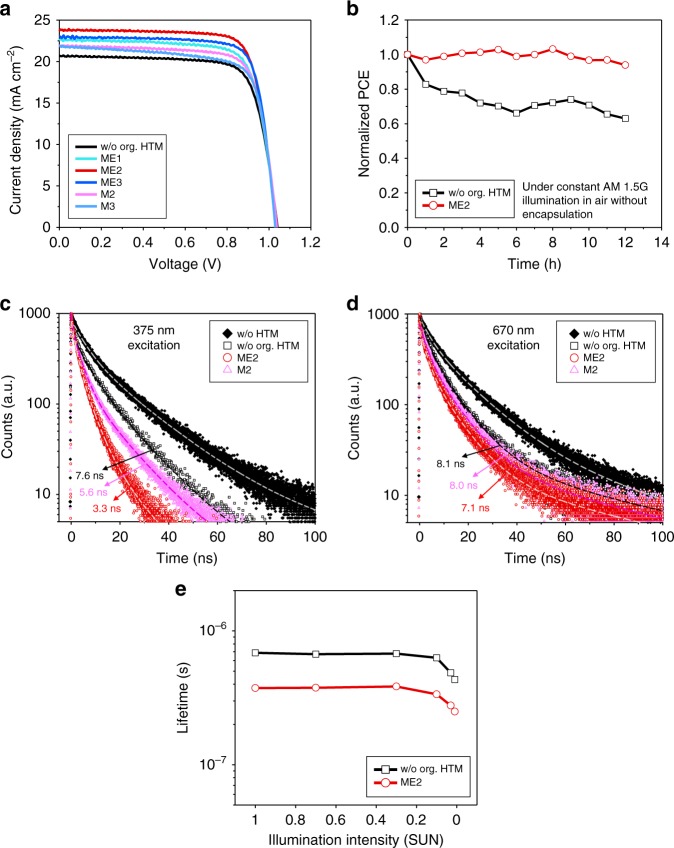
Performances of PSCs with organic HTMs. **a** *J*–*V* curves of PSCs with the designed organic HTMs (ITO/NiO_*x*_/organic HTM/perovskite/PCBM/ZnO/Al). All data were measured at AM 1.5G (100 mW cm^−2^ intensity). The classification of all colored lines is the same as that in Fig. 2 and black line represents the PSC without organic HTM. **b** Photostability tests under constant AM 1.5G illumination with a xenon lamp, including UV radiation, for two PSC devices without encapsulation in air (black line: ITO/NiO_*x*_/perovskite/PCBM/ZnO/Al and red line: ITO/NiO_*x*_/ME2/perovskite/PCBM/ZnO/Al). **c**, **d** Time-resolved photoluminescence decays of perovskite layers casted on various HTMs (black-filled diamond, black square, red circle and pink triangle symbols are for quartz/perovskite, quartz/NiO_*x*_/perovskite, quartz/NiO_*x*_/ME2/perovskite, and quartz/NiO_*x*_/M2/perovskite, respectively): **c** 375 and **d** 670 nm laser sources were utilized for excitation. **e** TPC measurement: charge extraction lifetime vs. illumination intensity plots of complete PSC structures measured at short-circuit current condition. Black and red lines are for ITO/NiO_*x*_/perovskite/PCBM/ZnO/Al and ITO/NiO_*x*_/ME2/perovskite/PCBM/ZnO/Al, respectively

The increased work function of anode by the dipole effect leads to the enhanced built-in electric field, consequently increasing short-circuit current density (*J*_sc_). Figure 5a and Table 2 confirm that the photo-generated current of PSC could be improved by adding ESIPT-active ME1 (6.03 D at K*) and it was further increased with ME2 having higher dipole moment (7.64 D at K*) as expected. However, we could notice the slightly decreased photocurrent from the PSC with ME3 having the highest dipole moment (9.64 D at K*), and this is due to the maximized effective conjugation length within the ME3 molecule, which not only increases its absorption efficiency but also shifts its absorption band to the visible wavelength region (Fig. 2a), consequently sacrificing the transmitted light into the perovskite photoactive layer. As for the non-proton-transfer compounds (M2 and M3), of which the transition dipole moment effect cannot be intensified, the photocurrent enhancement was not remarkable, but their relatively higher dipole moments on both ground and excited states than those of the conventional HTMs such as spiro-OMeTAD and PTAA were still efficient to provide better device performances. Meanwhile, as the applied bias increases toward *V*_oc_ condition, the built-in electric field within the device is compensated by the applied bias, and thus the induced dipole moment aligned with the electric field within the thin film would be weakened due to the minimized electric filed inside the device, which could not affect the work function of anode. KPFM results in Fig. 3i and Supplementary Fig. 29 clearly show that the light-driven potential differences of anode, observed at short-circuit condition and reverse bias condition, almost disappear under the forward bias condition approaching *V*_oc_. Because the dipole effect would be minimized at *V*_oc_ condition, the variation of *V*_oc_ is dominated by the HOMO level of HTLs. M3 (−5.31 eV), ME2 (−5.30 eV), and ME3 (−5.31 eV) having slightly smaller HOMO levels than M2 (−5.35 eV) and ME1 (−5.38 eV) showed a little bit lower *V*_oc_ (0.01 V).

**Table 2:** Parameters of best-performing PSCs with various organic HTMs

HTM	*V*_oc_ (V)	*J*_sc_ (mA cm^−2^)	FF	PCE (%)
w/o org. HTM	1.04	20.72	0.76	16.31
ME1	1.04	22.58	0.76	17.85
ME2	1.03	23.74	0.78	19.22
ME3	1.03	23.02	0.79	18.73
M2	1.04	21.98	0.76	17.37
M3	1.03	21.84	0.75	16.87
Spiro-OMeTAD	1.01	21.12	0.76	16.36
PTAA	0.99	21.27	0.72	15.16

*J*−*V* characteristics of our PSCs showed negligible hysteresis depending on scan direction (Supplementary Fig. 30) and rate (Supplementary Fig. 31), and the PCEs measured by scanning were well-matched to those obtained at the maximum power point (Supplementary Fig. 32), indicating the stable operation of our PSC devices. The statistical data of PSCs are given in Supplementary Table 2 and Supplementary Fig. 33. Additionally, ESIPT-active molecules can efficiently block the UV light into the perovskite photoactive layer, and we could observe the improved photostability under constant full-Sun illumination, including UV radiation (AM 1.5G, 100 mW cm^−2^, unencapsulated in air), even without any other strategies (Fig. 5b and Supplementary Fig. 34).

### Investigation on the effect of photo-excited HTM

The improved charge extraction property of photo-enhanced organic HTM through the ESIPT process could be proven by the PL decay signal difference of perovskite (e.g. an emission peak around 770 nm for perovskite), on the organic HTM, at two different light sources: 375 nm excitation that could excite both organic HTM and perovskite, and 670 nm excitation that could excite perovskite only, respectively. Figure 5c, d, Supplementary Table 3, and Supplementary Table 4 show that, at the 375 nm excitation, PL lifetime (*τ*_ave_) of perovskite on ESIPT-active ME2 (3.3×10^−9^ s) is much shorter than that of perovskite without organic HTM (7.6×10^−9^ s), but their difference is not remarkable at the 670 nm excitation (ME2: 7.1×10^−9^ s, w/o org. HTM: 8.1×10^−9^ s). This suggests that the ESIPT-active ME2 can significantly improve its charge extraction property at its excitation (e.g. solar illumination condition including UV light). Meanwhile, *τ*_ave_ variation of perovskite on M2, non-proton-transfer model compound of ME2, also shows the similar trend at different light sources, but its photo-enhancement effect is not as prominent as the ME2 case because its photo-excited state cannot be intensified.

**Fig. 6:**
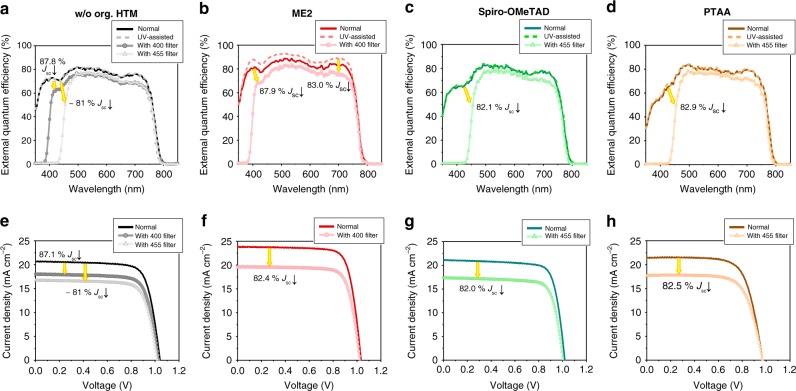
Characterization of the effect of photo-excited organic HTM on device performances. **a**–**d** External quantum efficiency (EQE) signals measured by the conventional method (solid line), UV-assisted method (dashed line), the conventional method with 400 nm cut-off filter (circle), and the conventional method with 455 nm cut-off filter (triangle): PSCs built on **a** NiO_*x*_, **b** NiO_*x*_/ME2, **c** NiO_*x*_/spiro-OMeTAD, and **d** NiO_*x*_/PTAA. **e**–**h** *J*–*V* curves measured at AM 1.5G 100 mW cm^−2^ intensity (solid line), at the same light condition with 400 nm cut-off filter (circle) and at the same light condition with 455 nm cut-off filter (triangle): PSCs built on **e** NiO_*x*_, **f** NiO_*x*_/ME2, **g** NiO_*x*_/spiro-OMeTAD, and **h** NiO_x_/PTAA. Percentage values in all plots represent the reduction ratio of *J*_sc_ after applying cut-off filters: **a**−**d** estimated by EQE and **e**–**h** measured by *J*–*V* characteristics

The external quantum efficiency (EQE) of solar cell device at the specific wavelength is calculated by measuring the extracted photocurrent under the monochromated light at that wavelength. However, the organic HTMs of *p−i−n* PSCs, usually having high bandgaps (mostly in UV region) for visible transparency, cannot be efficiently excited by the monochromated light in visible and near-IR region, and therefore the conventional incident photon-to-current efficiency (IPCE) measurement is not proper to represent the advantageous effect of photo-excited HTMs under solar irradiation to their EQE values in visible and near-IR range. To reflect this effect on their EQEs, the IPCE measurement system was modified as depicted in Supplementary Fig. 35. The PSC device was continuously exposed to the UV bias light using the additional light source to excite HTM, and the monochromated light at certain wavelength in interest was illuminated to the device for generating photocurrent at that wavelength. Finally, a modified-EQE value at that wavelength was calculated from the photocurrent, obtained by subtracting the UV bias light-generated photocurrent from the measured total current value at that wavelength (Fig. 6a–d). If the excitation of HTMs by the UV light would not induce any changes in their properties, the differences between the conventional and the UV-assisted EQE signals would be negligible. Figure 6a shows that the PSC without organic HTM has almost the same EQE signals from both measurement methods; however, ME2-applied PSC has much higher modified-EQE signal than that obtained from the conventional measurement (Fig. 6b), which supports that the charge extraction property of ESIPT-active HTM is improved by its photo-excitation. As for the PSCs having the conventional HTMs such as spiro-OMeTAD (Fig. 6c) and PTAA (Fig. 6d), EQE values from those two measurements are almost identical, which means that those HTMs do not have the photo-excitation-driven additional charge extraction property under broadband solar irradiation.

The performance enhancement of photo-excited ESIPT HTM can be further confirmed by the variation of *J*−*V* characteristics of PSC, especially the *J*_sc_, according to the application of an additional filter to the solar-simulated spectrum during the measurement, which can cut off the light enabling the organic HTM to be excited (Fig. 6e–h). Figure 6e, f shows the *J*_sc_ variation of PSC without an organic HTM and that with the best-performing ME2 HTM, respectively, before and after applying the filter, which cut off the solar spectrum below around 400 nm wavelength. *J*−*V* characteristics of the PSCs without an organic HTM provides 87.1 ± 0.8% decreased *J*_sc_ values after applying the cut-off filter (around 400 nm) (Fig. 6e), and this is well-matched to the reduction ratio of *J*_sc_, estimated by the EQE spectrum in Fig. 6a (87.8%); ratio of the conventional EQE-estimated *J*_sc_, measured with the filter, to that, measured without the filter. However, the decrease of *J*_sc_ in *J*−*V* characteristics of ME2-utilized PSCs depending on the application of the filter (82.4 ± 1.1%, Fig. 6f) is remarkably lower than the aforementioned ratio of the EQE-calculated *J*_sc_ values (87.9%, Fig. 6b), and this is rather well-correlated to the reduction ratio of *J*_sc_, calculated by the conventionally measured EQE with the filter, to *J*_sc_, calculated by the UV-assisted EQE without the filter (83.0%, Fig. 6b). This further supports that the photocurrent improvement of ESIPT-HTM-applied PSC, shown in the *J*−*V* characteristics, is triggered by the photo-excitation of HTM from the UV light in solar irradiation. For reference, *J*−*V* characteristics of ME2-based PSC, measured with the filter, did not show any hysteresis depending on the scan direction and rate, as shown in Supplementary Fig. 36. The similar studies were also conducted for the PSCs with the conventional HTMs such as spiro-OMeTAD and PTAA. In those HTM cases, a filter cutting off the light below around 455 nm was utilized to exclude photo-excitation effect, because their absorption bands encroach upon the visible wavelength region. As shown in Fig. 6c, d, g h, they showed the similar behaviors to the PSCs without an organic HTM. We could observe 82.0 ± 1.1% (Fig. 6g) and 82.5 ± 1.8% (Fig. 6h) decreased *J*_sc_ from the *J*−*V* characteristics of spiro-OMeTAD and PTAA with the cut-off filter, respectively, and they were well-matched to the conventional EQE-estimated *J*_sc_ reduction ratios (spiro-OMeTAD: 82.1% in Fig. 6c, PTAA: 82.9% in Fig. 6d), which means that those HTMs do not have the intensified photo-excited state effect.

Because the ESIPT-active HTMs developed in this work can emit the light between 400 and 600 nm wavelength range, which the perovskite layer can efficiently absorb for the charge generation, after absorbing the light within the UV region, it could be suspected that the enhanced *J*_sc_ values shown in the devices with ESIPT-active materials are from the so-called down-shifting effect of HTMs. However, firstly, the portion of the UV light in the solar simulator spectrum is not considerable, so the amount of the emitted light between 400 and 600 nm range, converted from the UV light, cannot be high enough to noticeably improve the *J*_sc_ shown in ESIPT-HTM devices, even though the quantum yields of the HTMs are high. Moreover, if there would be an increased photocurrent from that effect, it should be also shown in the EQE signals (measured by the conventional IPCE system) between 300 and 370 nm range, in which the absorption of ESIPT-materials is highest, but the difference of EQEs in this region between the device without organic HTM and that with ME2 is negligible (Supplementary Fig. 37). Additionally, although the emission from the HTMs between 400 and 600 nm range could be increased during the measurement of the modified-EQE signal due to the additional UV light source, the photocurrent obtained by the UV bias light was subtracted from the measured total current, and so it cannot affect the calculated modified-EQE signals. Consequently, the down-shifting effect of the designed HTMs cannot account for a noticeable portion of the observed improvement of *J*_sc_ values of the devices.

Meanwhile, the improved charge extraction and the reduced recombination properties of the best-performing ME2-applied PSC in actual device structure could be further confirmed by transient photocurrent (TPC), measured at short-circuit condition^67^. Figure 5e shows that the PSC with ME2 has much shorter extraction lifetimes than that without ME2 at each illumination intensity (from 0.01 to 1 Sun illumination). The shorter extraction lifetime would be beneficial to suppress the charge recombination and to enhance the charge extraction in device architectures.

## Discussion

HTLs of *p−i−n* planar PSCs are continuously exposed to light during the operation under broadband solar irradiation, and therefore their property at the photo-excited state could be an important factor to be considered for designing efficient HTMs. We have especially focused on their transition dipole moment variation at their photo-excitation, and firstly developed a molecular design principle to intensify this effect for the high-performance organic HTMs of *p−i−n* PSCs. In detail, several useful approaches, which can be additionally considered after satisfying basic prerequisites for HTM, are suggested for designing: (i) control of the effective conjugation length of asymmetric molecular structure providing high transition dipole moment without sacrificing the transmitted light to the perovskite and (ii) maximization of the photo-excited-state lifetime of organic HTM through the ESIPT process to preserve its high dipole moment. The presented molecular design principle is readily applicable to many organic HTM developments for PSCs. Our results showed that the photo-enhanced organic HTMs were advantageous to reduce the charge recombination and to improve the charge extraction, consequently increasing the photocurrent of PSCs. Additionally, their UV-blocking ability was beneficial to improve the photostability of PSCs. We expect that the strategic combination of the developed HTM design rules, rationally devised molecular structures for the enhanced transition dipole moment at photo-excited state with the increased lifetime, and the characterization of the photo-excitation-driven performance improvement of the PSC devices could be an effective option to be considered for designing highly efficient HTMs of PSCs.

## Methods

### Materials for synthesis

All the reagents and anhydrous solvents were purchased from Aldrich, Alfa Aesar, and Acros Organics and used as received without further purification. All the glasswares, syringes, magnetic stirrer bars, and needles were thoroughly dried in a convection oven before use. Silica gel column chromatography was performed with silica gel 60 (particle size 0.0063 to 0.200 mm, Merck).

### General synthetic procedure

*Synthesis of bromo-imidazole derivatives*: The bromo-imidazole derivatives were synthesized by the classical method of lophine synthesis in high yields. To a stirred solution of *α*-diketone (1.0 *eq*.), aniline (1.0 *eq*.), and ammonium acetate (3.0 *eq*.) in acetic acid, aldehyde (1.0 *eq*.) was added at room temperature. Then, the reaction mixture was refluxed overnight. After completion of the reaction, the reaction mixture was cooled down to room temperature and quenched into copious amount of water. The pH was neutralized with *aq*. sodium bicarbonate solution. The precipitated crude products were filtered and washed with water, and dried using anhydrous magnesium sulfate in dichlomethane. Silica gel column chromatography was performed using *n*-hexane and ethyl acetate (9:1 v/v ratio) as eluent to obtain pure imidazole derivatives (1, 2, 3, and 4 in Supplementary Figs. 1 and 2) (*Y* = between 68 and 70 %).

*Phenoxazine-substituted imidazole derivatives*: To a solution of bromo-imidazole derivatives (1, 2, 3, and 4 in Supplementary Figs. 1 and 2) (1.0 *eq*.) in toluene, phenoxazine (1.1 *eq*.), palladium (II) acetate (0.033 *eq*.), and potassium *t-*butoxide (3.3 *eq*.) dissolved in toluene were added. Then, a solution of tri-*tert*-butylphosphine (0.12 *eq*.) in toluene was added, and the mixture was stirred and heated under reflux condition for 12 h. After the completion of the reaction, the mixture was cooled down to room temperature and poured into copious amount of water. The crude product was extracted using chloroform and the organic layers were washed with brine. Then, the organic layer was separated and dried with anhydrous magnesium sulfate. Silica gel column chromatography was performed using *n*-hexane and chloroform (4:1 v/v ratio) as eluent to obtain pure phenoxazine-substituted imidazole derivatives (ME1, ME2, ME3, and M3) (*Y* = between 64 and 67%). As for M2, 1.0 *eq*. of ME2 was dissolved in *N,N′*-dimethylformamide at room temperature. 2.0 *eq*. of K_2_CO_3_ and 2.0 *eq*. of iodomethane were added sequentially, and then the mixture was stirred under dark condition for 12 h. After the completion of the reaction, the reaction mixture was poured into water and pH was neutralized with *aq*. sodium bicarbonate solution. After extraction of the crude product with dichlomethane, the product was dried using anhydrous magnesium sulfate. Silica gel column chromatography was performed using *n*-hexane and ethyl acetate (3/1 v/v ratio), and white M2 powder was obtained quantitatively (*Y* = around 98%).

### Characterization of organic molecules

^1^H NMR and ^13^C NMR spectra were characterized by Bruker 400 MHz NMR spectrometer (Bruker-DRX400), and the chemical shifts (*δ*) and coupling constant (*J*) were expressed in ppm and hertz, respectively. Mass spectra were obtained by mass spectrometer JEOL (JMS-AX505WA), and elemental analysis was performed using CE Instrument (EA1110) instrument. Thermal analyses of DGA/TGA were performed by SDT Q600 V20.9 Build 20 instrument. Cyclic voltametric experiments were executed with a model 273A (Princeton Applied Research) using a one-compartment electrolysis cell consisting of a platinum working electrode, a platinum wire counter electrode, and a quasi Ag+/Ag electrode as a reference. The measurements were carried out in 0.5 mM CH_2_Cl_2_ solution with tetrabutylammonium tetrafluoroborate as supporting electrolyte at a scan rate of 50 mV s^−1^. Each oxidation potential was calibrated with ferrocene as a reference.

### Computational details

The geometry optimization and quantum chemical calculation including dipole moments of ME1, ME2, ME3, M2, M3, spiro-OMeTAD, and PTAA were performed by DFT/B3LYP (Becke’s three-parameter exchange function and Lee−Yang−Parr correlation function) method with 6-31G(d, p) basis sets^49,50^. Time-dependent DFT^68^ was used with 6-31G(d, p) to obtain the electronic transition energies that include some accounts of electron correlation. All the calculations were carried out using Gaussian 09 program package^69^.

### PSC device fabrication

Acetone, isopropyl alcohol, and deionized water were sequentially used to clean the patterned ITO-coated glass substrates using ultrasonication. The oxygen plasma treatment (for 5 min) was utilized before using the ITO substrates to improve the wettability of the following solution. NiO_*x*_ nanoparticle solution (20 mg ml^−1^) was spin-cast on the ITO layer (2000 rpm for 20 s) and annealed at 150 °C for 10 min. The procedure for the NiO_*x*_ solution preparation is described elsewhere^70^. ME1, ME3, and M3 were dissolved in chlorobenzene, and ME2 and M2 were dissolved in pyridine without additives. The organic HTM solution (10 mg ml^−1^) was spin-cast on NiO_*x*_ layer at 2000 rpm for 30 s and annealed at 120 °C for 15 min. The bottom portion of organic HTM was partially mixed with NiO_*x*_ nanoparticle layer. The thicknesses of organic HTMs were 30−40 nm, measured by atomic force microscopy (AFM) and SEM. We could not find significant thickness change of organic HTMs after casting perovskite solution. To control the thickness of organic HTM, glass/organic HTM/perovskite stacks were additionally prepared. A cross-sectional SEM image of one of the glass/organic HTM/perovskite samples is shown in Supplementary Fig. 27b. For spiro-OMeTAD and PTAA layers, the following solutions were prepared: spiro-OMeTAD (35 mg) was dissolved in chlorobenzene (1 ml) with Li-bis(trifluoromethanesulphonyl)imide (4.41 mg), acetonitrile (17.5 µl) and 4-tert-butylpyridine (14.4 µl), and PTAA (*M*_n_ = 17,500 g mol^−1^, 10 mg) was dissolved in toluene (1 ml) with Li-bis(trifluoromethanesulphonyl)imide (1.275 mg), acetonitrile (7.5 µl) and 4-tert-butylpyridine (4 µl). The perovskite solution (CH_3_NH_3_PbI_3_) was prepared by dissolving CH_3_NH_3_I and PbI_2_ (Sigma Aldrich) 1:1 molar ratio in the solvent mixture of dimethylsulfoxide and *γ*-butyrolactone (3:7 v/v) for a total concentration of 1.3 M in an N_2_ atmosphere. The solution was stirred at 70 °C for at least 6 h before use. The perovskite layer was formed onto the HTM by a consecutive two-step spin-casting process at 1000 and 5000 rpm for 10 and 20 s, respectively. During the second spin-casting step, the substrate was treated with toluene drop. The substrate was dried on a hot plate at 100 °C for 10 min. PCBM (20 mg ml^−1^ in chlorobenzene) was spin-cast on the perovskite layer as an electron collection and transport layer, and 2.5 wt% ZnO nanoparticle (NP) dispersion solution (nanograde, N-10) was further spin-cast on top of the PCBM layer. Finally, samples were moved into a thermal evaporator and Al was thermally deposited at a base pressure of 4×10^−6^ Torr.

### PSC device characterization

The current density−voltage (*J*−*V*) characteristics have been measured using a class AAA solar simulator (Wacom: WXS-220S-L2), which has a xenon lamp and three halogen lamps for a high precision under AM 1.5G illumination (100 mW cm^−2^), at a 0.05 V s^−1^ scan rate in both forward and backward direction. During the test, the stage temperature was kept at 25 °C by using a chiller. The light intensity was calibrated with an NREL-certified Si photodiode equipped with an infrared cut-off filter (KG5) to reduce spectral mismatch, and the active area of PV cell was defined using an opaque metal mask (0.1024 cm^2^) to reduce the influence of the scattered light. The EQE was measured at short-circuit condition using an ABET Technology 10500 solar simulator as the light source and a SPECTRO Mmac−200 as the light solution. For UV-assisted EQE, UV light source having 300−400 nm range (centered at 365 nm) and 4 W power was utilized.

### Fluorescence anisotropy and KPFM measurement

Fluorescence anisotropy measurements were conducted by fluorometer (Shimazu, F7000, Japan) with a polarizer, which has adequate wavelength range (300−700 nm). The excitation light was polarized along the *z*-axis by a vertically installed polarizer. Incident beam was set to a convenient wavelength (375 nm) to trigger the transition dipole of various organic layers. We obtained the emission of polarized organic layers when the incident and the emitted light are both polarized along the *z*-axis (*I*_║_) and the emitted light is polarized in a perpendicular direction with respect to the exciting radiation (*I*_⊥_). Parallel (*I*_║_) and perpendicular (*I*_⊥_) emission of the polarized excited beam were exploited to calculate the fluorescence anisotropy as *r* = (*I*_║_ − *I*_⊥_)/(*I*_║_ + 2*GI*_⊥_). In our experiments *G* value was calibrated to be 1.4. To verify the fluorescence anisotropy decrease by the electric field, the following structure was utilized: ITO/NiO_*x*_/organic HTM/ZnO/Al. KPFM was performed with a commercial AFM machine (NX10, Park Systems). KPFM images were taken in a noncontact mode with a PPP-EFM tip. And the custom-made vertical sample holder was used to measure the cross-section of the samples.

### Organic FET

SiO_2_/Si (300 nm SiO_2_/n-doped Si) substrates were cleaned by sonication in acetone and isopropyl alcohol for 20 min for each in sequence. After fully drying in a nitrogen stream, the substrates were further cleaned by exposing them to 360 nm UV light for 15 min. After then, ME2 (or ME3) layer was prepared by spin-coating method using 5 mg ml^−1^ solution. On top of the ME2 (or ME3) layer, Al_2_O_3_ layer (20 nm) was deposited by atomic layer deposition at 100 °C with a deposition rate of 1.3−1.6 Å cycle^−1^ and pentacene layer was thermally deposited under a vacuum of 2.0×10^−6^ Torr at a deposition rate of 0.4−0.5 Å s^−1^. Finally, top-contact gold source/drain electrodes were thermally deposited under a vacuum of 2.0×10^−6^ Torr at a deposition rate of 0.1−0.2 Å s^−1^. The channel lengths (*L*) and width (*W*) were 150 μm and 1 mm, respectively. The current−voltage characteristics of OFETs were measured using Keithley 4200 semiconductor parameter analyzer connected to a probe station. Illumination of the OFETs was carried out by using a Xe lamp light source with a monochromator that was connected to a probe station system through a light guiding optical fiber.

### TRPL and TPC measurement

TRPL curves were obtained using a commercial TCSPC system (FluoTime 200, PicoQuant). Samples were excited by using a picosecond diode laser of 670 nm (LDH-P-C-670, PicoQuant) and 375 nm (LDH-P-C-375, PicoQuant) with a repetition rate of 4 MHz. The emitted PL was collected using a fast photomultiplier tube detector (PMA 182, PicoQuant) with a magic angle (54.7°) arrangement. The incident angle of excitation pulse was set to be about 30° with respect to the sample. The resulting instrumental response function was about 160 ps in full-width-half-maximum. The PL decays were measured at the emission peak (770 nm) for perovskite. Cut-off filters for 670 nm (FF01-692 nm, Semrock) and 375 nm (Stock #84-754, Edmund Optics) were applied to block the remaining scattering from the incident beam. For PL decays of organic HTMs, 375 nm excitation was utilized. TPC measurement was conducted by generation of a small perturbed bias utilizing a nanosecond OPO laser system (INDI-40-10, Spectra-Physics) from Nd:YAG laser and a background illumination from Xe lamp (LS-150-XE ABET). The devices were located inside a jig directly connected to a digital oscilloscope (DSO-X 3054A, Agilent), and 50 Ω input impedance was installed for short-circuit conditions for transient current. The bias light intensity was controlled precisely by wheel type of neutral density filter (NDC-100C-4M) for attenuating the peak intensity. The initial light intensity from the Xe lamp was adjusted using power meter to be equivalent to 1 Sun (100 mA cm^−2^). Whenever the filter was set up for decreasing light from the lamp, measurements of attenuated light from laser pulse of 550 nm through ND filter were verified using a laser power meter. We also controlled the peak current of the transient to be kept at below 10% of the steady-state current.

## Electronic supplementary material

Supplementary Information file

## Data Availability

The authors declare that the data supporting the findings of this study are available within the paper and the Supplementary Information, as well as from the corresponding authors upon reasonable request.
