# Supplementary material for: Management of transition dipoles in organic hole-transporting materials under solar irradiation for perovskite solar cells
Source: Nat Commun. 2018 Oct 31;9:4537. doi: 10.1038/s41467-018-06998-1 (PMC6208393; doi:10.1038/s41467-018-06998-1)
Supplement: Supplementary file 1 — Supplementary Information file [file 41467_2018_6998_MOESM1_ESM.pdf]

Supplementary Information

Management of transition dipoles in organic hole-transporting materials under solar irradiation for perovskite solar cells

Song Ah Ok,[1,†] Bong Hyun Jo,[2,†] Sivaraman Somasundaram,[3,†] Hwi Je Woo,[4] Dae Woon Lee,[5] Zijia Li,[2] Bong-Gi Kim,[6] Jong H. Kim,[5] Young Jae Song,[4,7,8,9] Tae Kyu Ahn,[2] Sanghyuk Park[3,*] & Hui Joon Park[1,10,*]

[1] Department of Energy Systems Research, Ajou University, Suwon 16499, Korea

[2] Department of Energy Science, Sungkyunkwan University, Suwon 16419, Korea

[3] Department of Chemistry, Kongju National University, Kongju 32588, Korea

[4] SKKU Advanced Institute of Nanotechnology (SAINT), Sungkyunkwan University, Suwon 16419, Korea

[5] Department of Molecular Science and Technology, Ajou University, Suwon 16499, Korea

[6] Department of Organic and Nano System Engineering, Konkuk University, Seoul 05029, Korea

[7] Center for Integrated Nanostructure Physics, Institute for Basic Science (IBS), Sungkyunkwan University, Suwon, 16419, Korea

[8] Department of Physics, Sungkyunkwan University, Suwon, 16419, Korea

[9] Department of Nano Engineering, Sungkyunkwan University, Suwon, 16419, Korea

[10] Department of Electrical and Computer Engineering, Ajou University, Suwon 16499, Korea

*Corresponding Authors: huijoon@ajou.ac.kr, spark0920@kongju.ac.kr

[†]S.A.O., B.H.J, and S.S. contributed equally to this work.

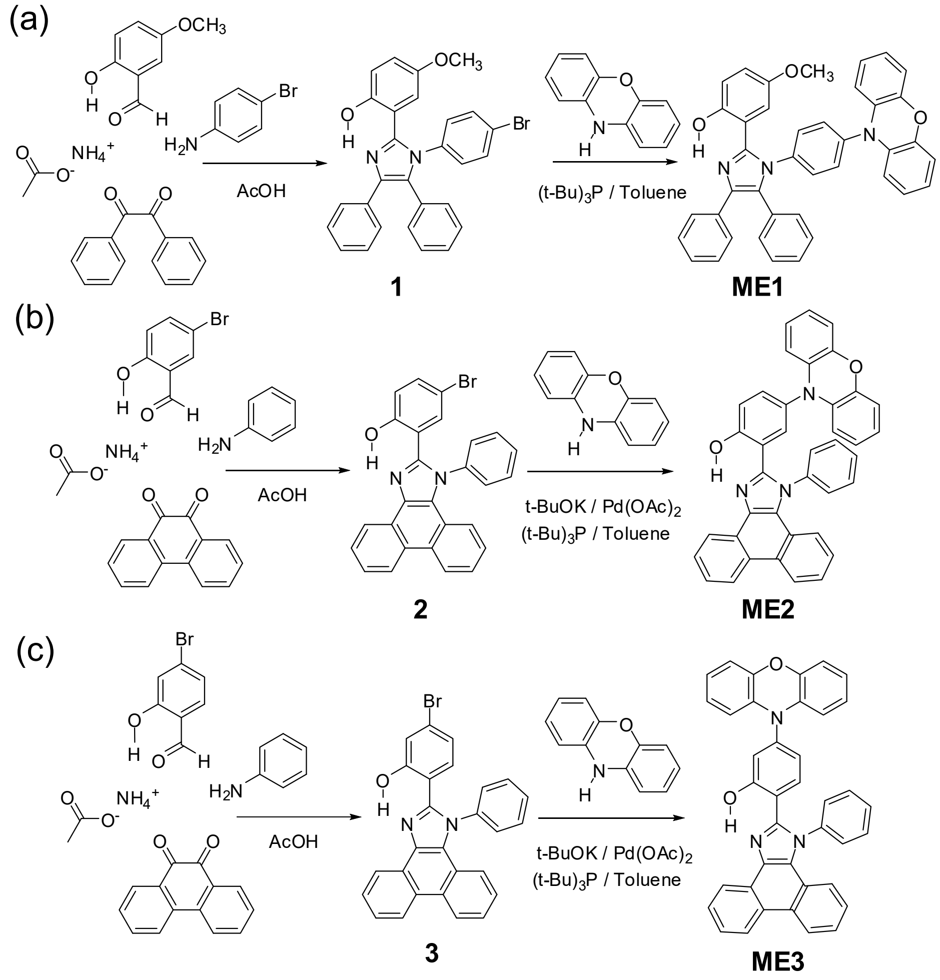

Supplementary Figure 1 | Chemical structures and detailed synthetic routes of ESIPT-molecules. **a**, ME1. **b**, ME2. **c**, ME3.

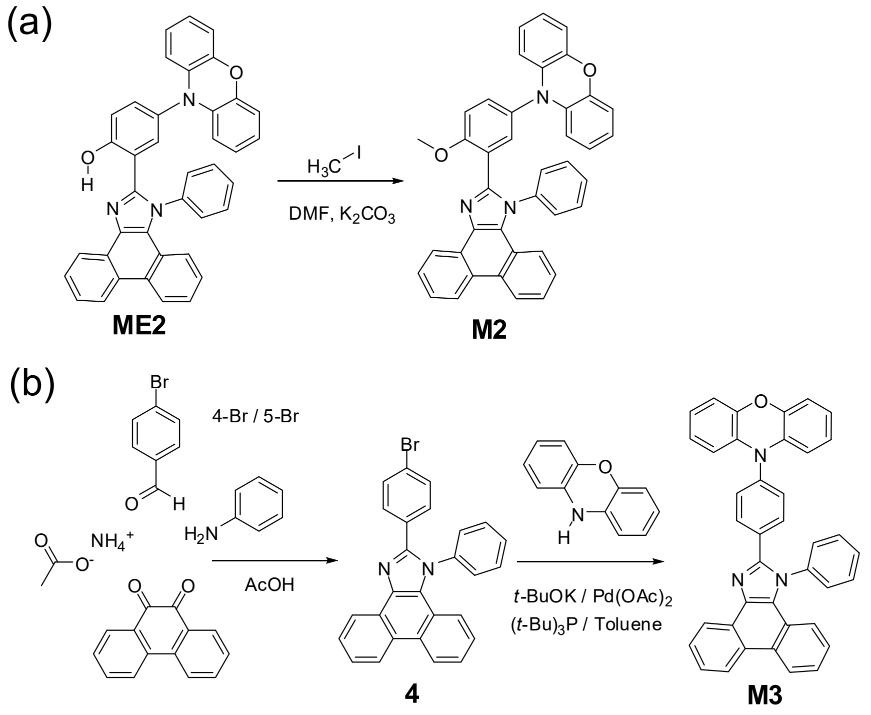

Supplementary Figure 2 | Chemical structures and detailed synthetic routes of non-proton-transfer model compounds. **a**, M2. **b**, M3.

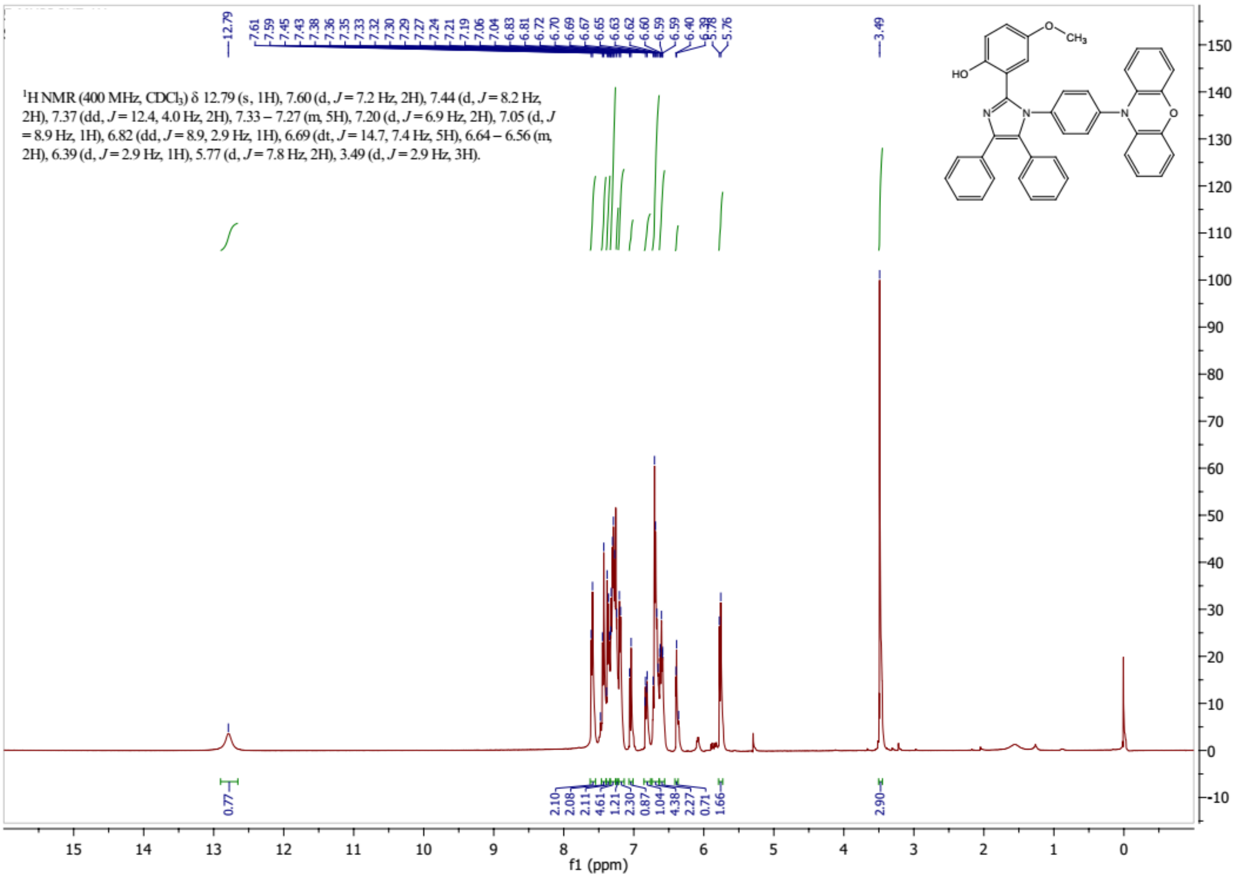

Supplementary Figure 3 | ^{1}H NMR of 2-(1-(4-(10H-phenoxazin-10-yl)phenyl)-4,5-diphenyl-1H-imidazol-2-yl)-4-methoxyphenol (ME1).

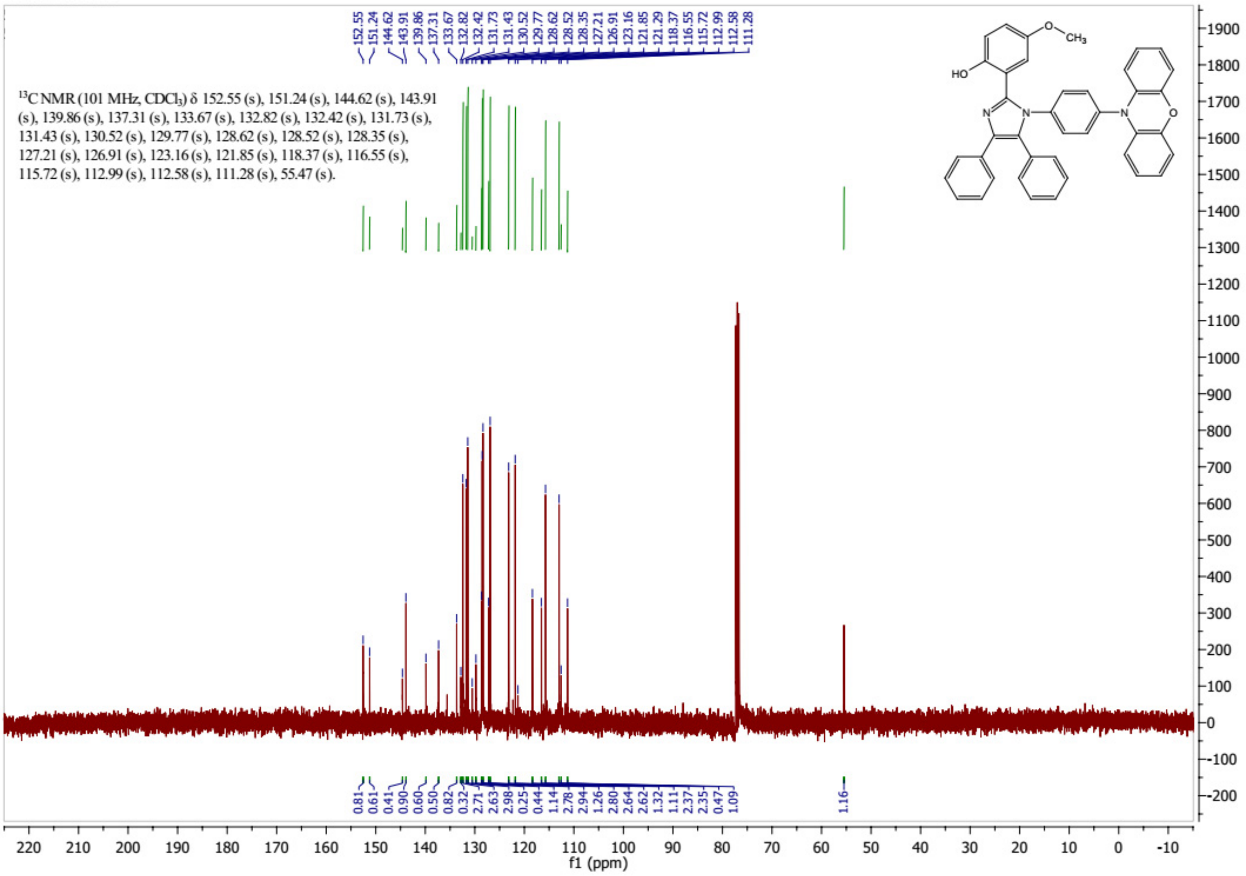

Supplementary Figure 4 | ^{13}C NMR of 2-(1-(4-(10H-phenoxazin-10-yl)phenyl)-4,5-diphenyl-1H-imidazol-2-yl)-4-methoxyphenol (ME1).

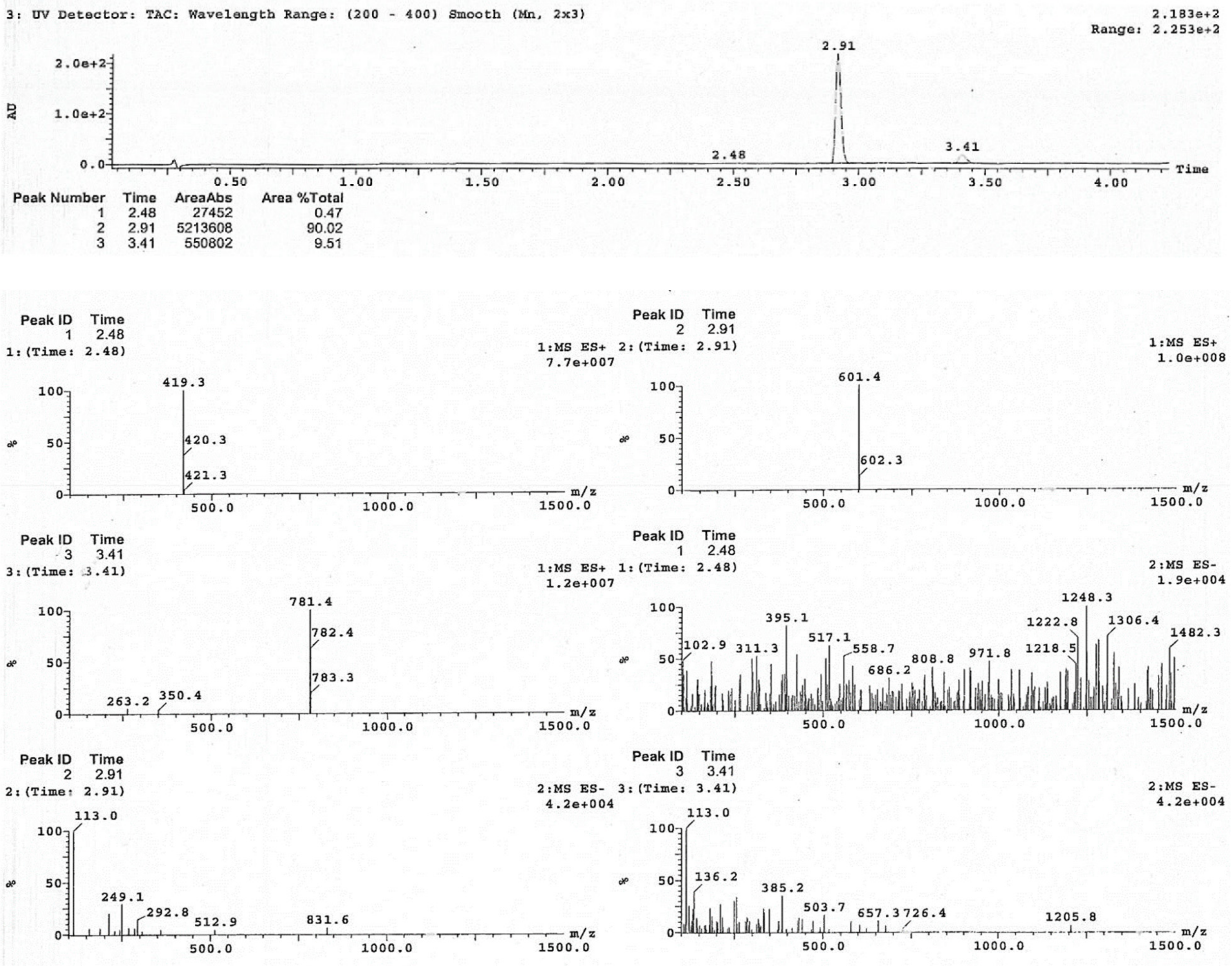

Supplementary Figure 5 | LC-MS analysis results of 2-(1-(4-(10H-phenoxazin-10-yl)phenyl)-4,5-diphenyl-1H-imidazol-2-yl)-4-methoxyphenol (ME1).

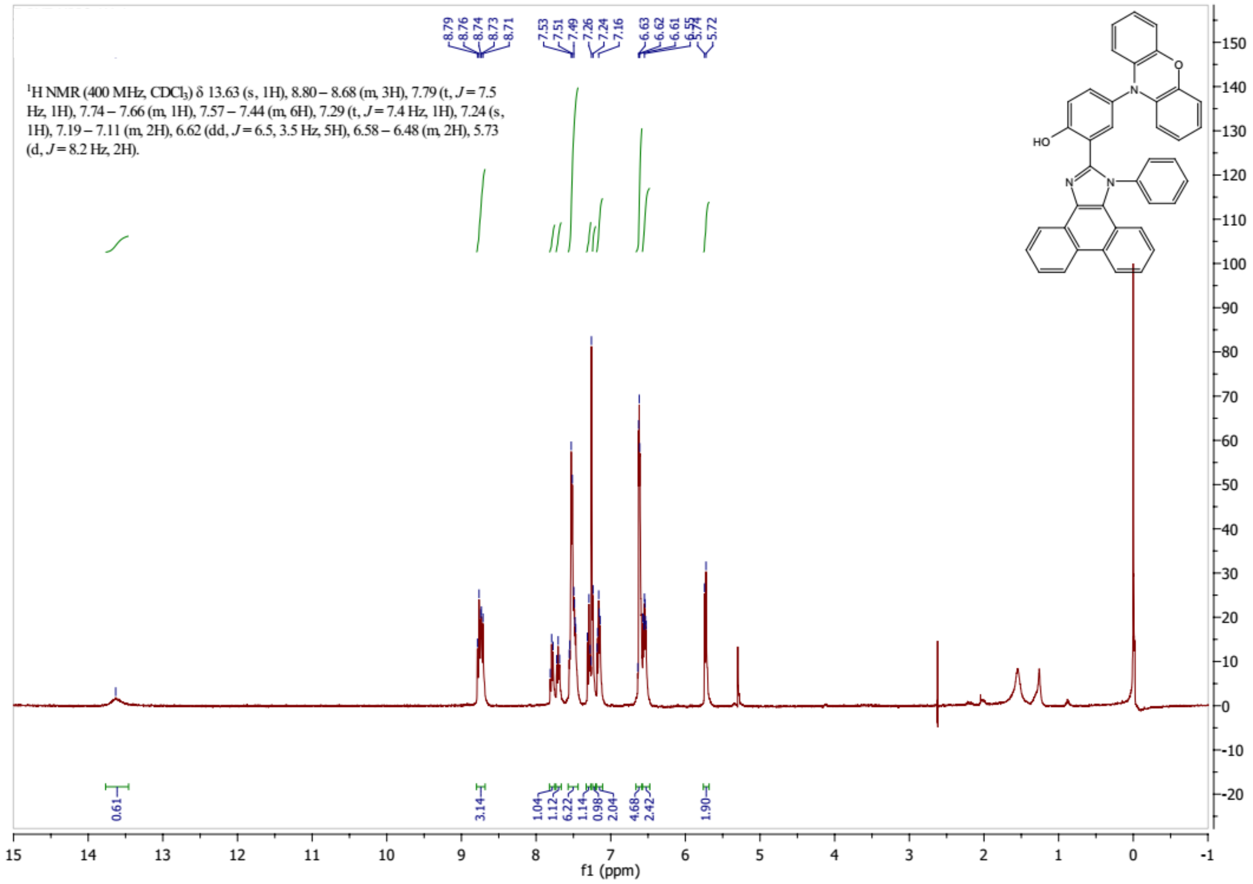

Supplementary Figure 6 | ^{1}H NMR of 4-(10H-phenoxazin-10-yl)-2-(1-phenyl-1H-phenanthro[9,10-d]imidazol-2-yl)phenol (ME2).

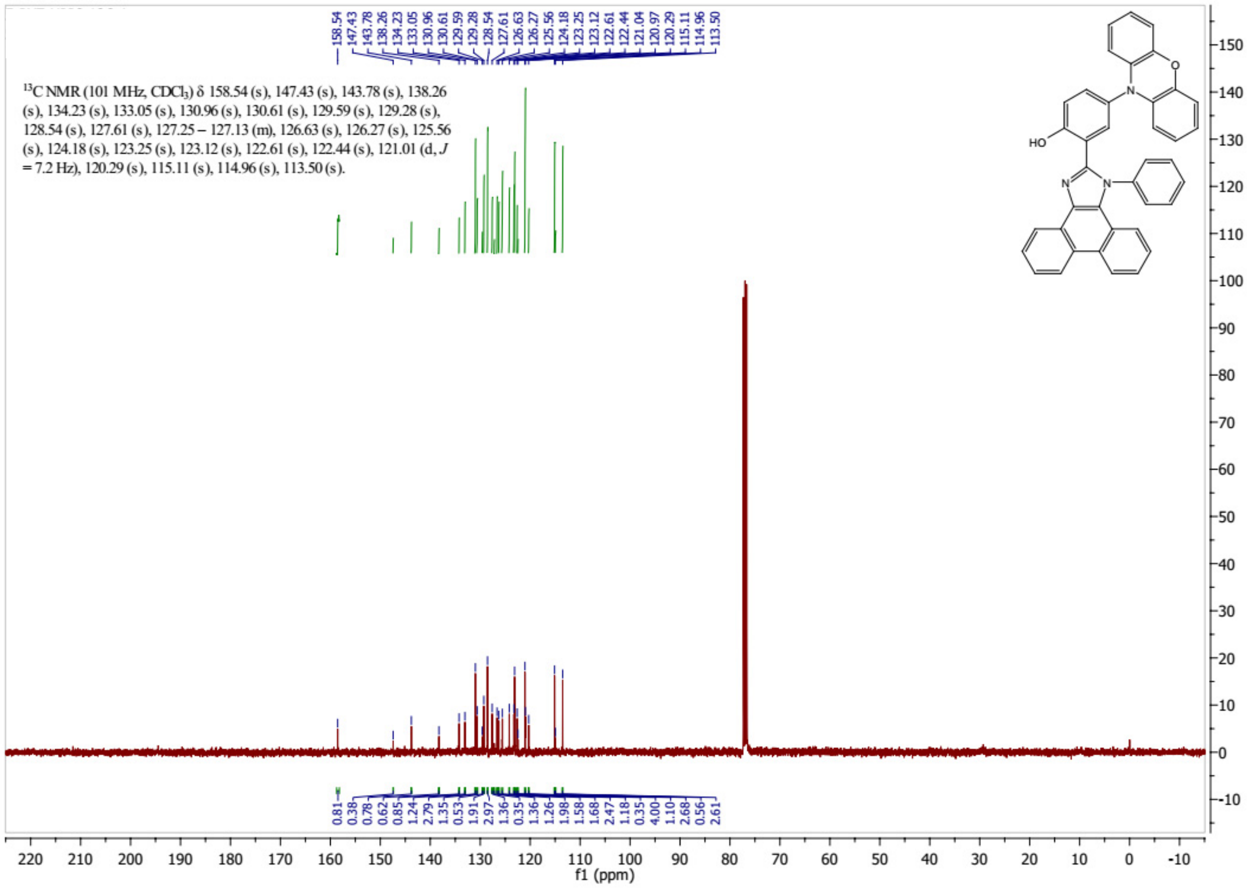

Supplementary Figure 7 | ^{13}C NMR of 4-(10H-phenoxazin-10-yl)-2-(1-phenyl-1H-phenanthro[9,10-d]imidazol-2-yl)phenol (ME2).

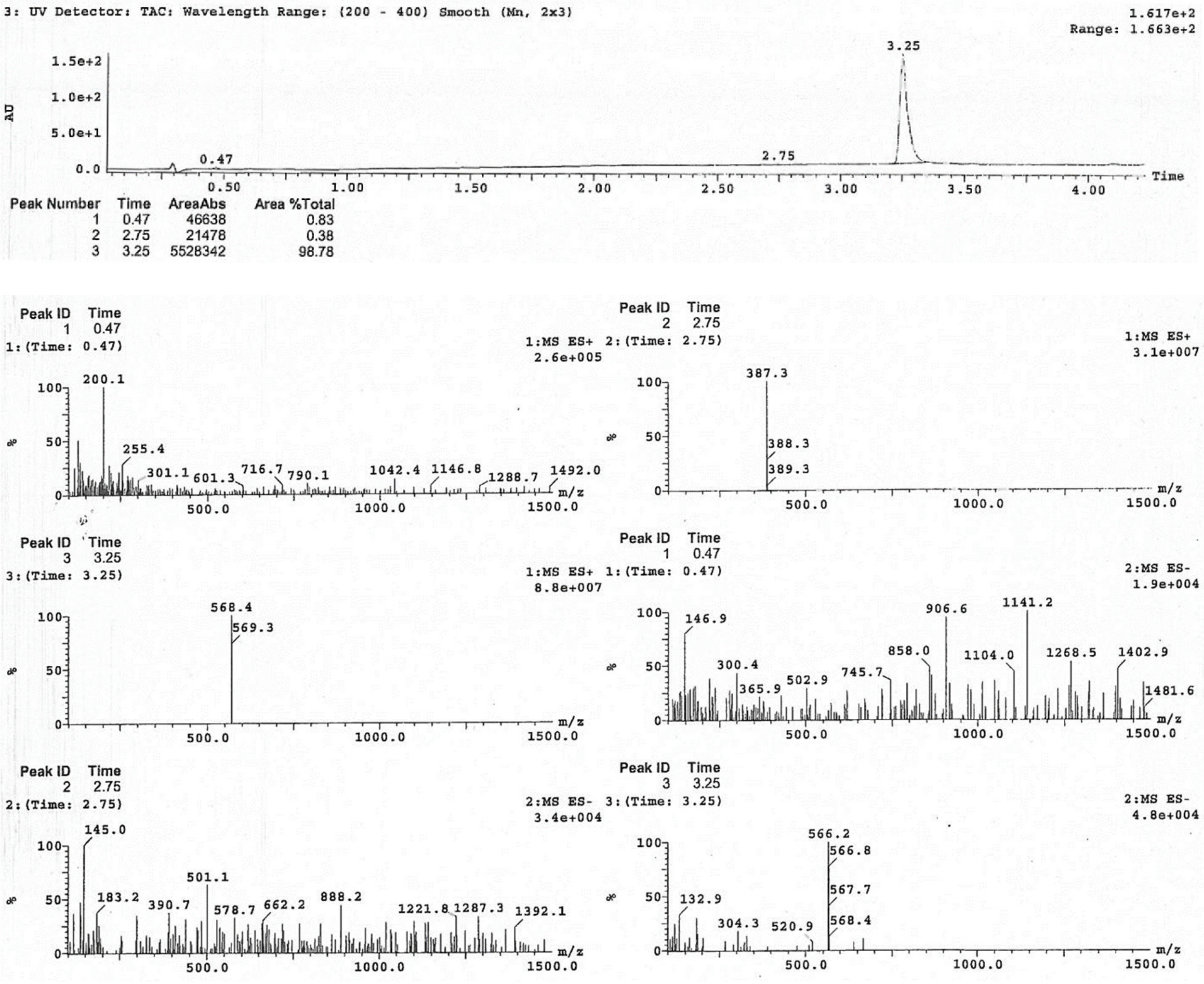

Supplementary Figure 8 | LC-MS analysis results of 4-(10H-phenoxazin-10-yl)-2-(1-phenyl-1H-phenanthro[9,10-d]imidazol-2-yl)phenol (ME2).

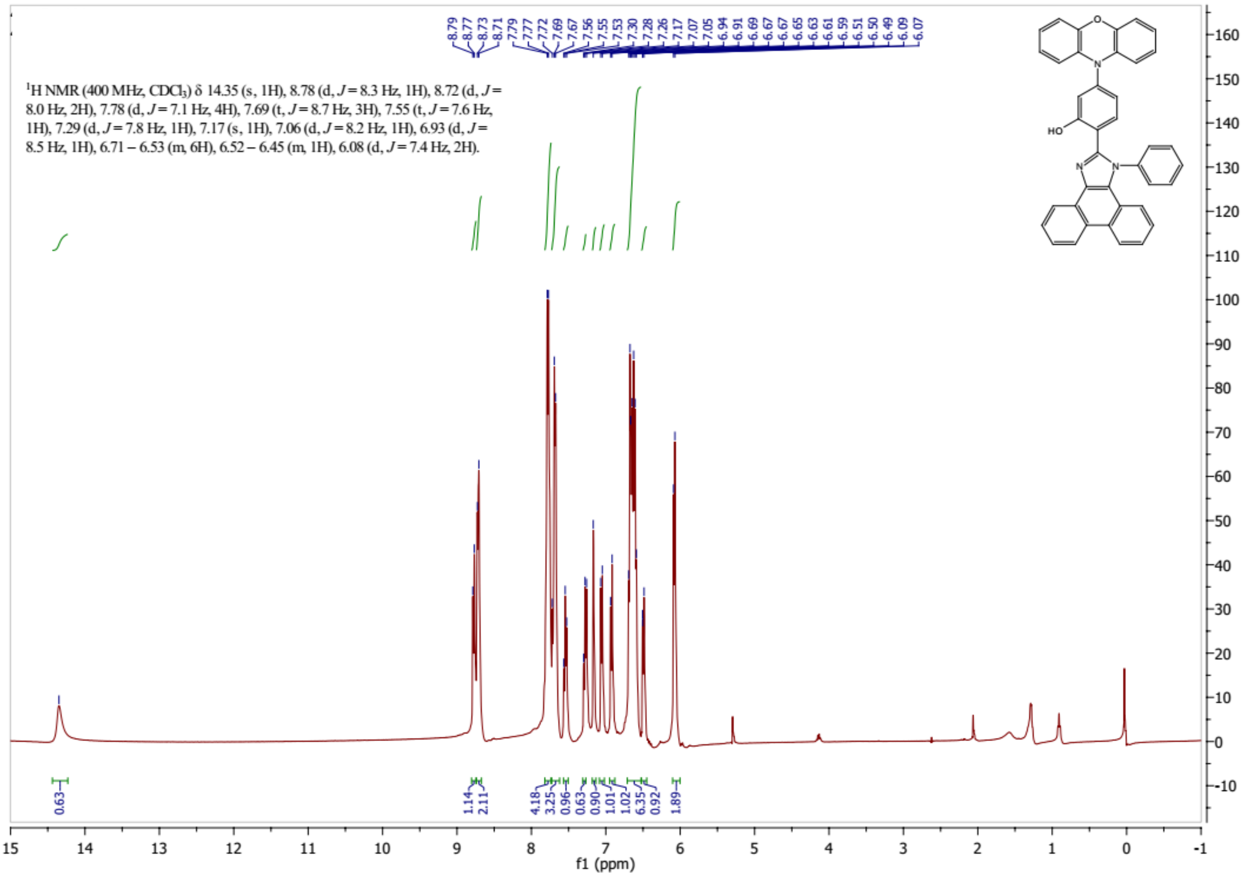

Supplementary Figure 9 | ^{1}H NMR of 5-(10H-phenoxazin-10-yl)-2-(1-phenyl-1H-phenanthro[9,10-d]imidazol-2-yl)phenol (ME3).

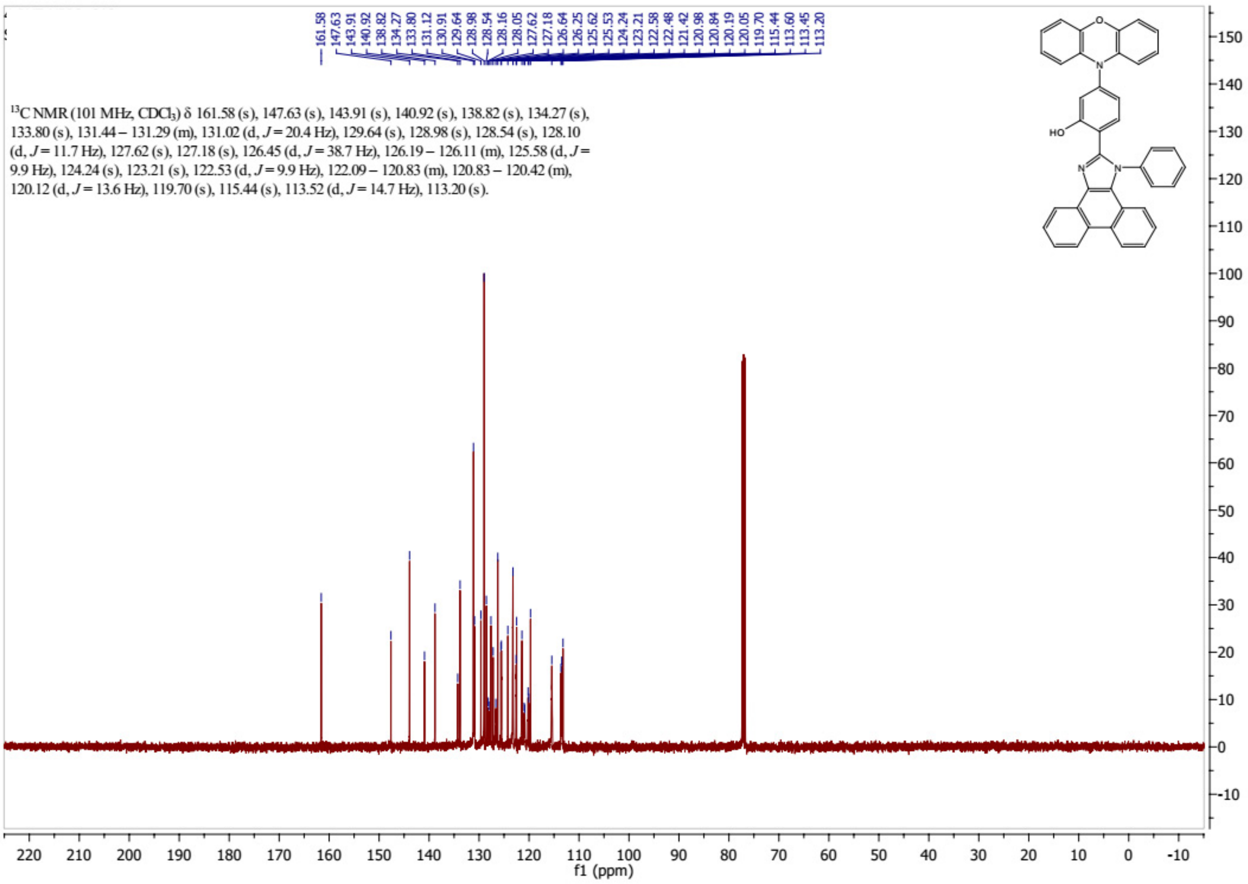

Supplementary Figure 10 | ^{13}C NMR of 5-(10H-phenoxazin-10-yl)-2-(1-phenyl-1H-phenanthro[9,10-d]imidazol-2-yl)phenol (ME3).

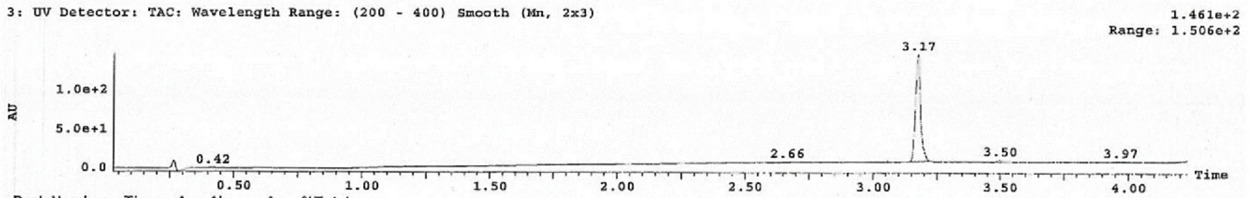

Peak Number	Time	AreaAbs	Area %Total
1	0.42	19429	0.58
2	2.66	14260	0.42
3	3.17	3235570	95.90
4	3.50	67443	2.00
5	3.97	37070	1.10

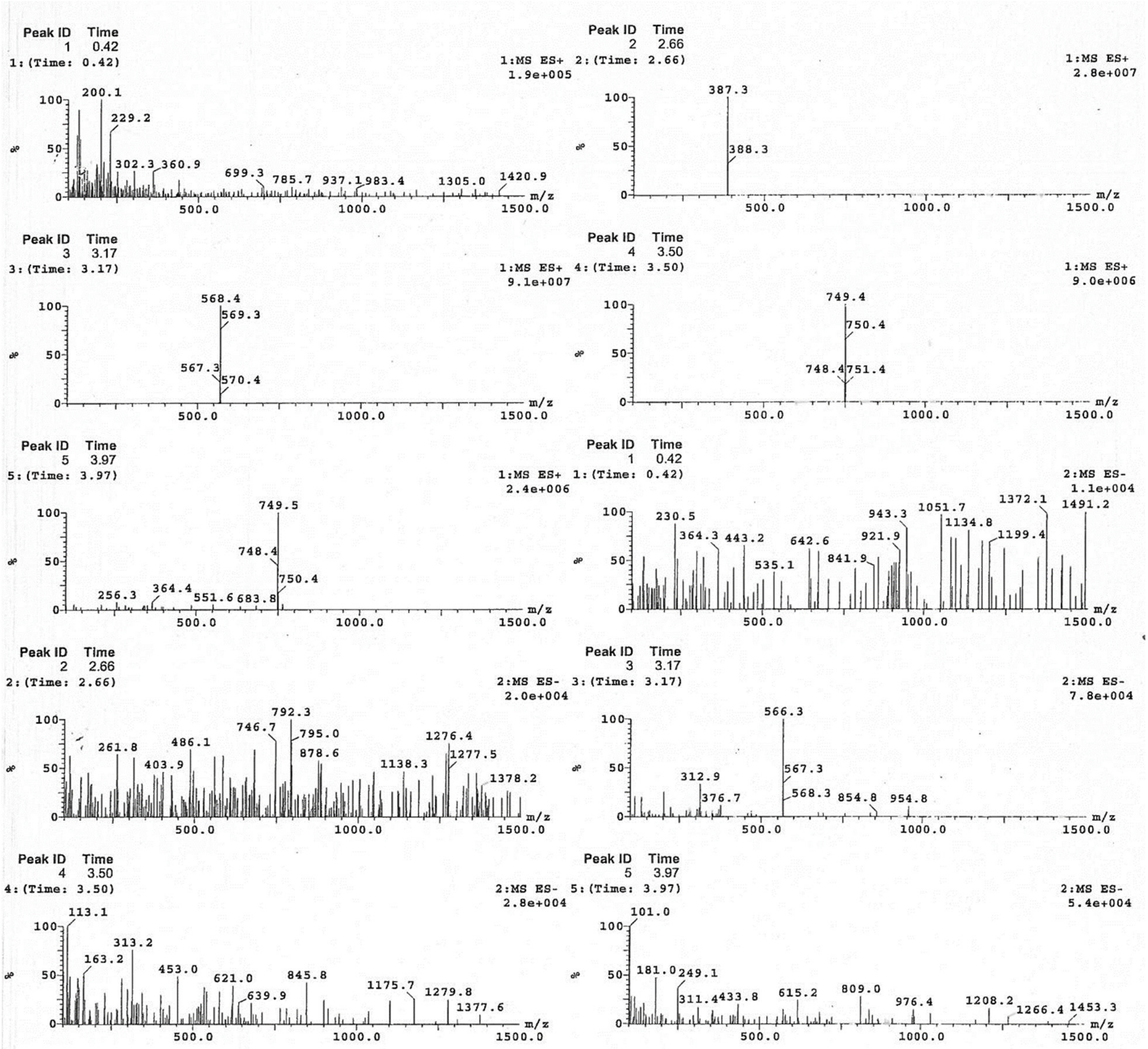

Supplementary Figure 11 | LC-MS analysis results of 5-(10H-phenoxazin-10-yl)-2-(1-phenyl-1H-phenanthro[9,10-d]imidazol-2-yl)phenol (ME3).

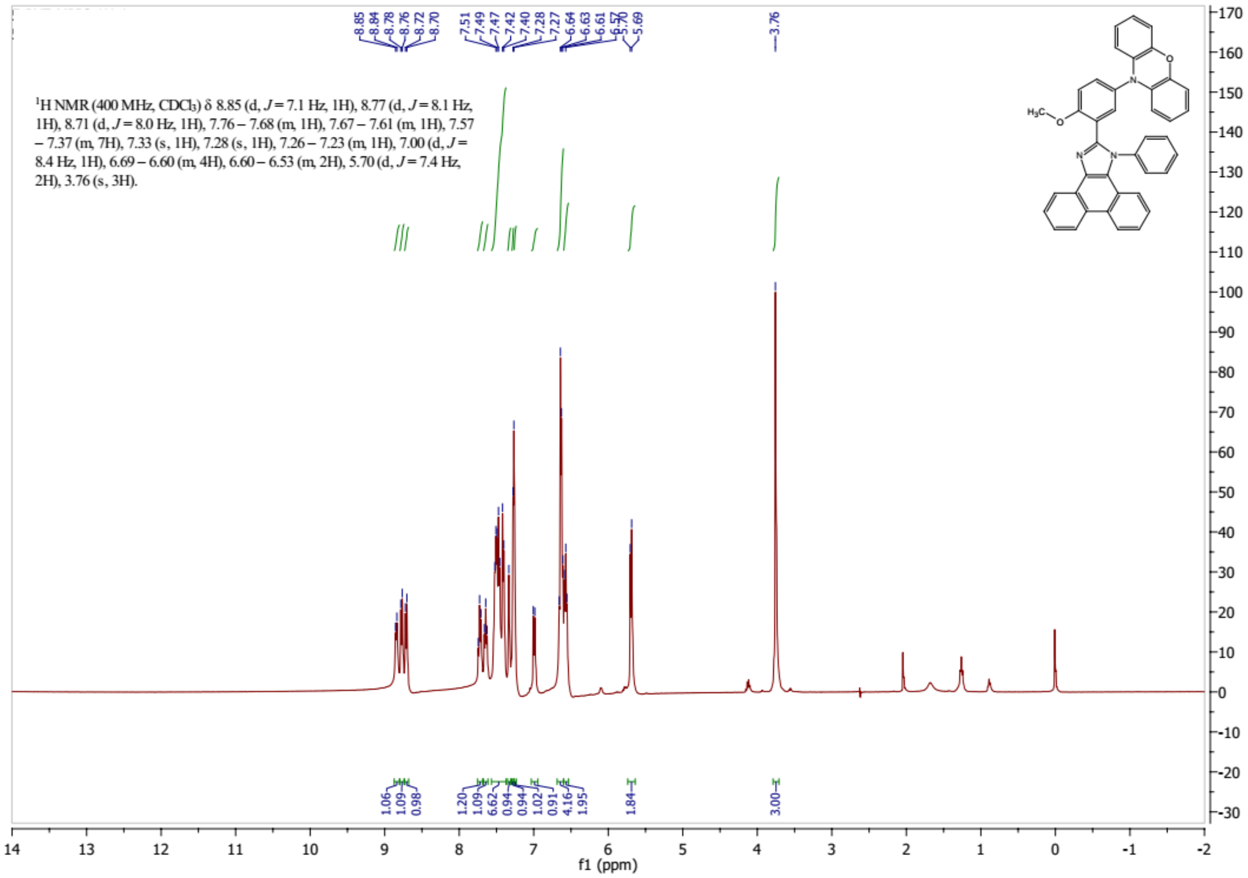

Supplementary Figure 12 | ^{1}H NMR of 10-(4-methoxy-3-(1-phenyl-1H-phenanthro[9,10-d]imidazol-2-yl)phenyl)-10H-phenoxazine (M2).

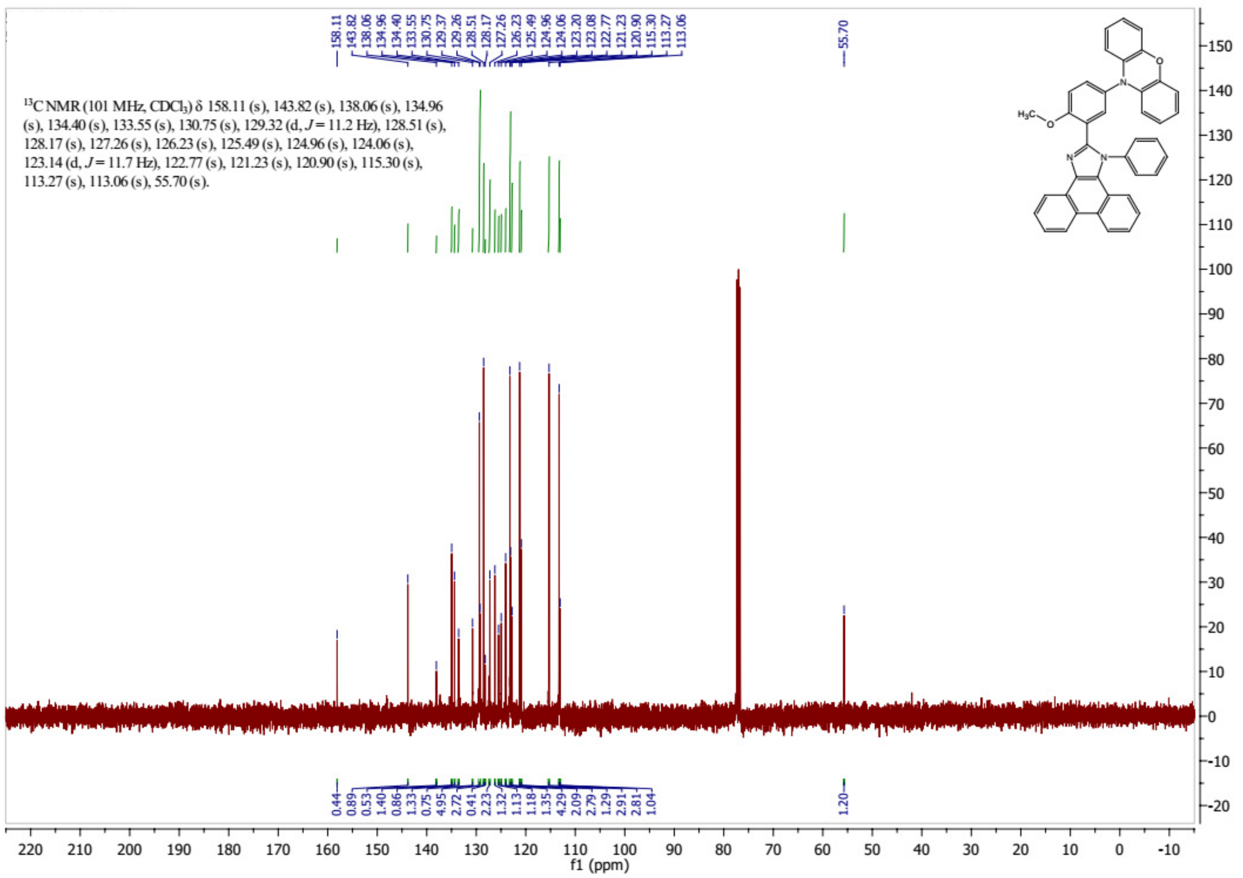

Supplementary Figure 13 | ^{13}C NMR of 10-(4-methoxy-3-(1-phenyl-1H-phenanthro[9,10-d]imidazol-2-yl)phenyl)-10H-phenoxazine (M2).

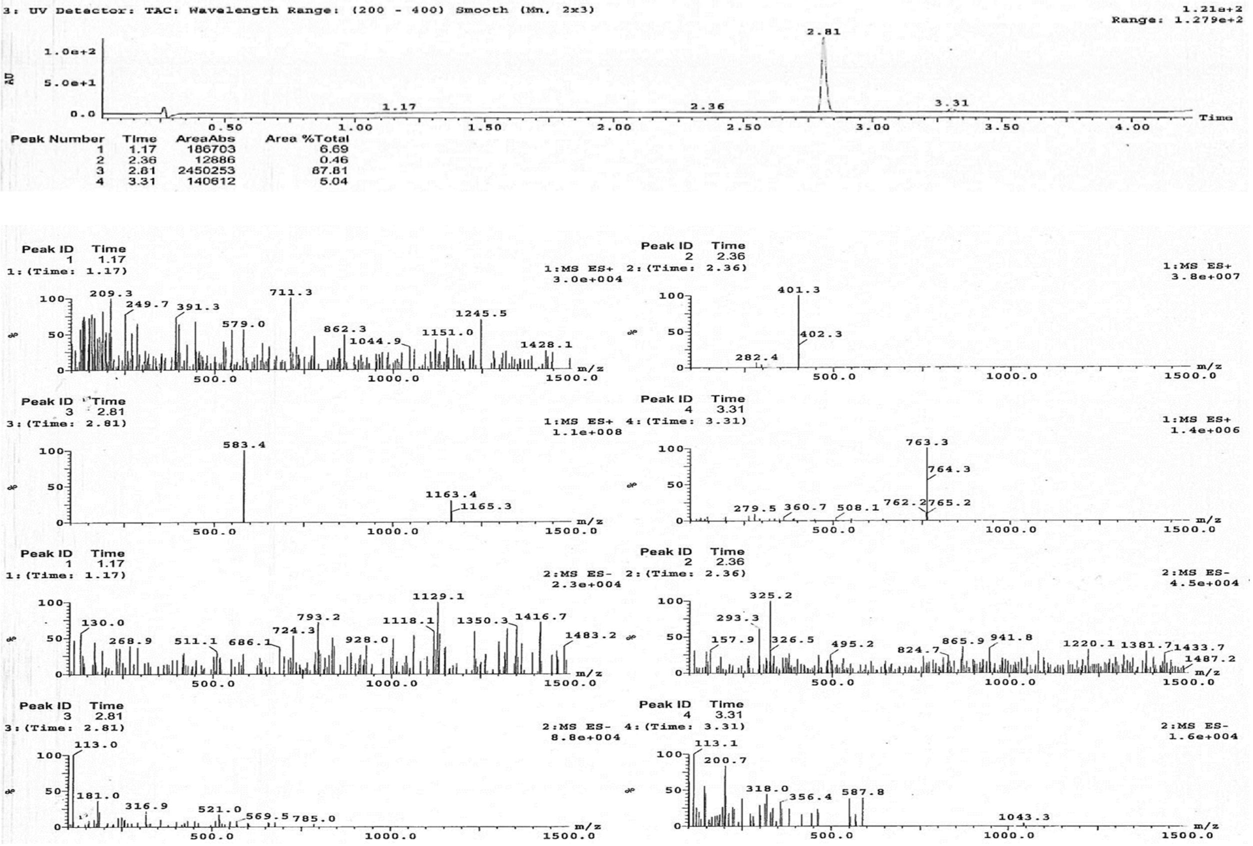

Supplementary Figure 14 | LC-MS analysis results of 10-(4-methoxy-3-(1-phenyl-1H-phenanthro[9,10-d]imidazol-2-yl)phenyl)-10H-phenoxazine (M2).

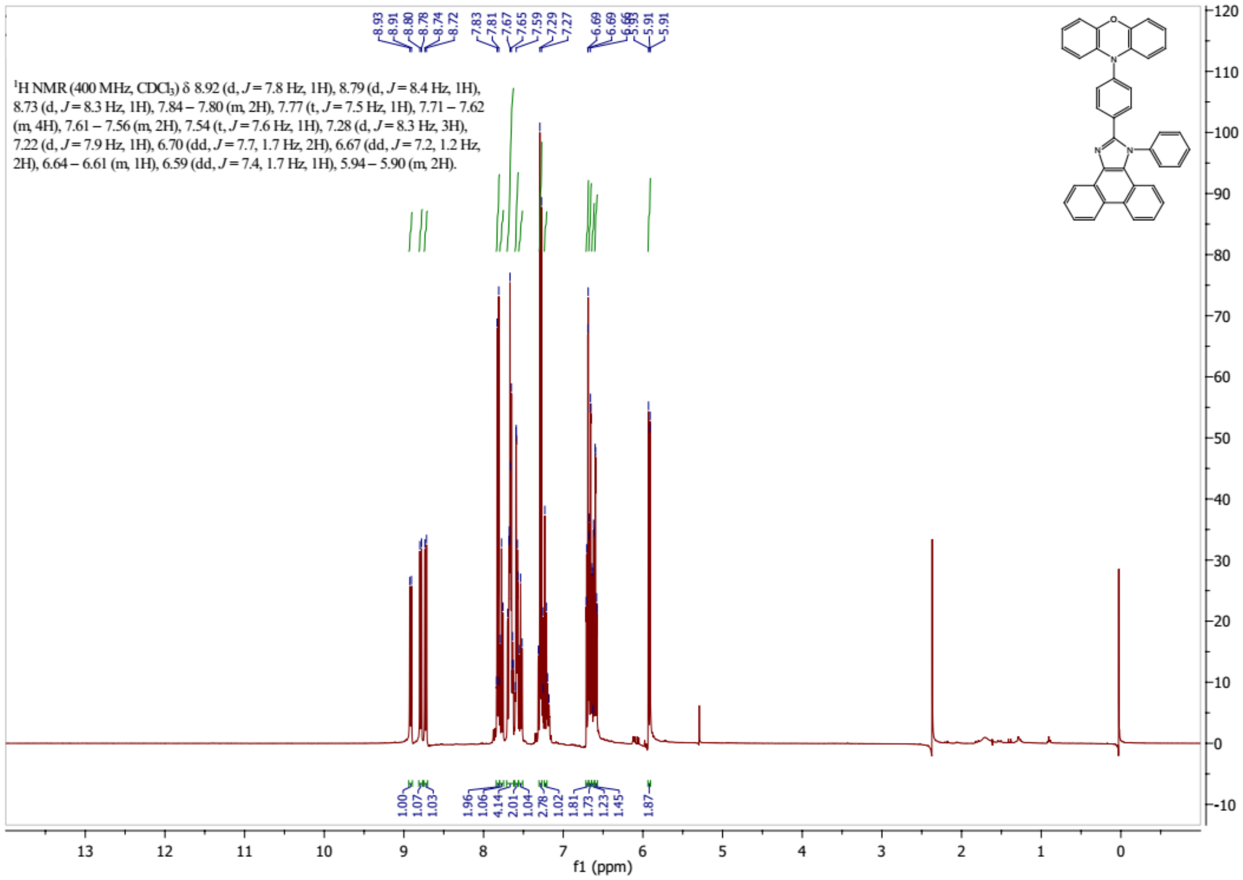

Supplementary Figure 15 | ^{1}H NMR of 10-(4-(1-phenyl-1H-phenanthro[9,10-d]imidazol-2-yl)phenyl)-10H-phenoxazine (M3).

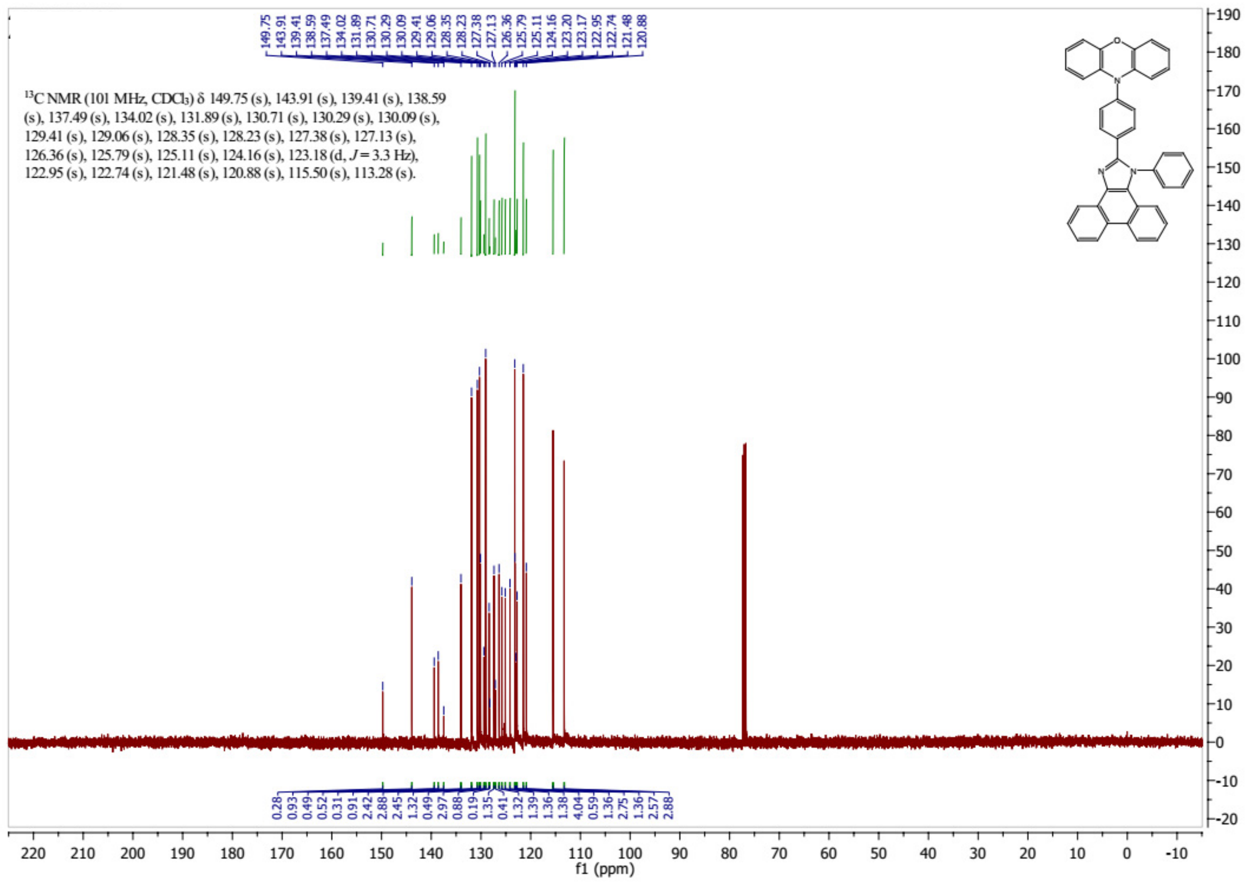

Supplementary Figure 16 | ^{13}C NMR of 10-(4-(1-phenyl-1H-phenanthro[9,10-d]imidazol-2-yl)phenyl)-10H-phenoxazine (M3).

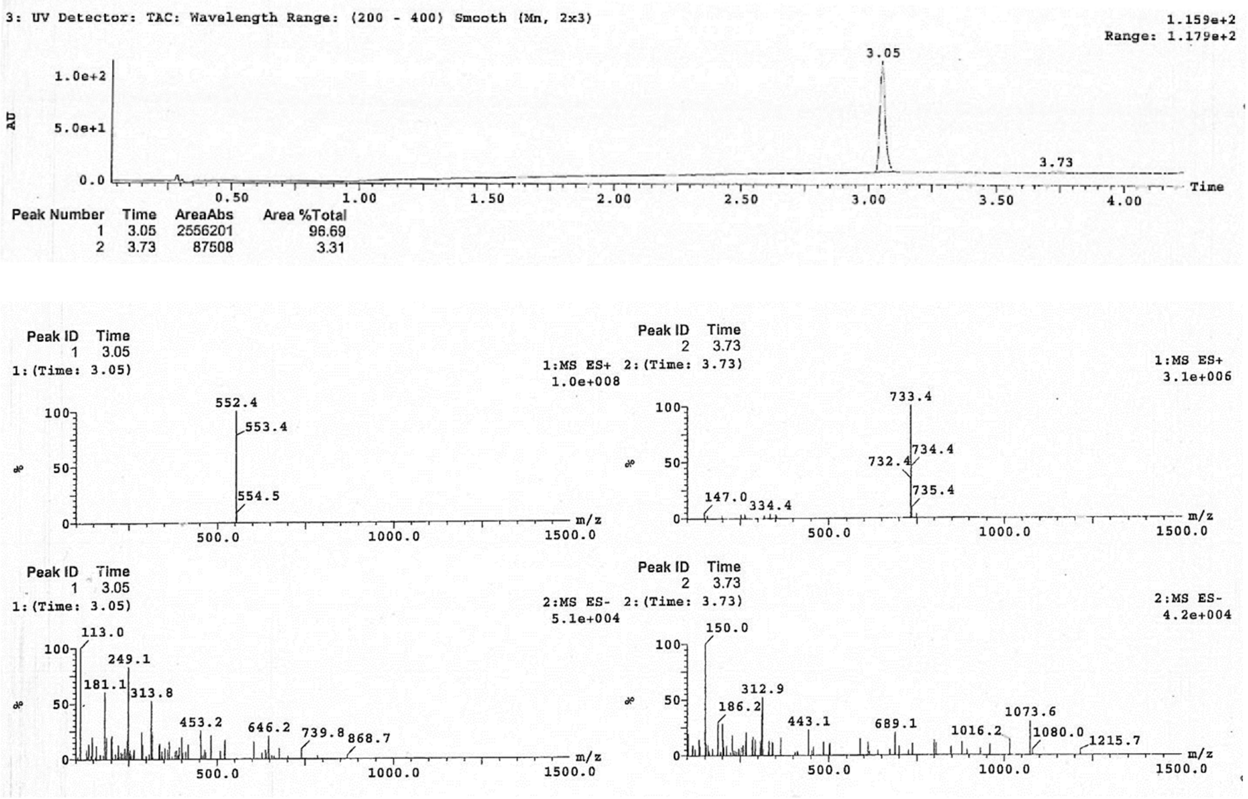

Supplementary Figure 17 | LC-MS analysis results of 10-(4-(1-phenyl-1H-phenanthro[9,10-d]imidazol-2-yl)phenyl)-10H-phenoxazine (M3).

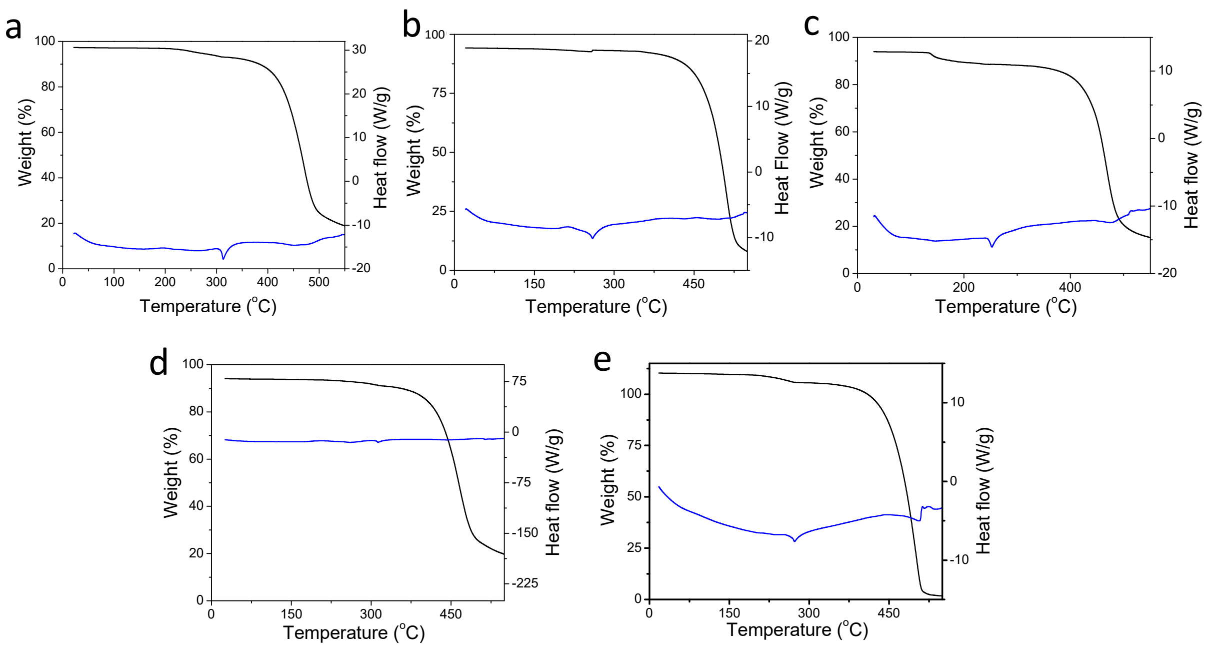

Supplementary Figure 18 | DSC (blue) and TGA (black) traces of **a**, ME2, **b**, ME3, **c**, ME1, **d**, M2, and **e**, M3.

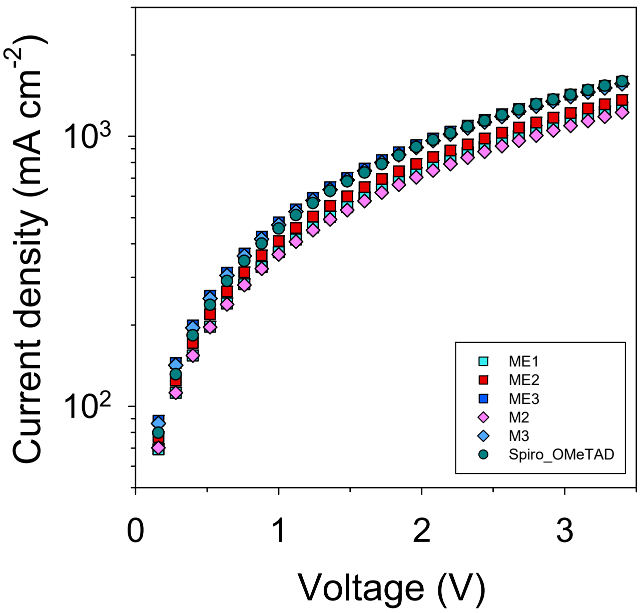

Supplementary Figure 19 | Measured *J–V* plots under dark condition for hole-only devices (ITO/NiO_x/organic HTM/MoO_3/Al). The calculated hole mobility values of ME1, ME2, ME3, M2, M3, and spiro-OMeTAD from a space-charge limited current (SCLC) method are 4.5×10^{-5}, 5.0×10^{-5}, 5.9×10^{-5}, 4.4×10^{-5}, 5.6×10^{-5}, and 5.8×10^{-5} $cm^2 \cdot V^{-1} \cdot s^{-1}$, respectively.

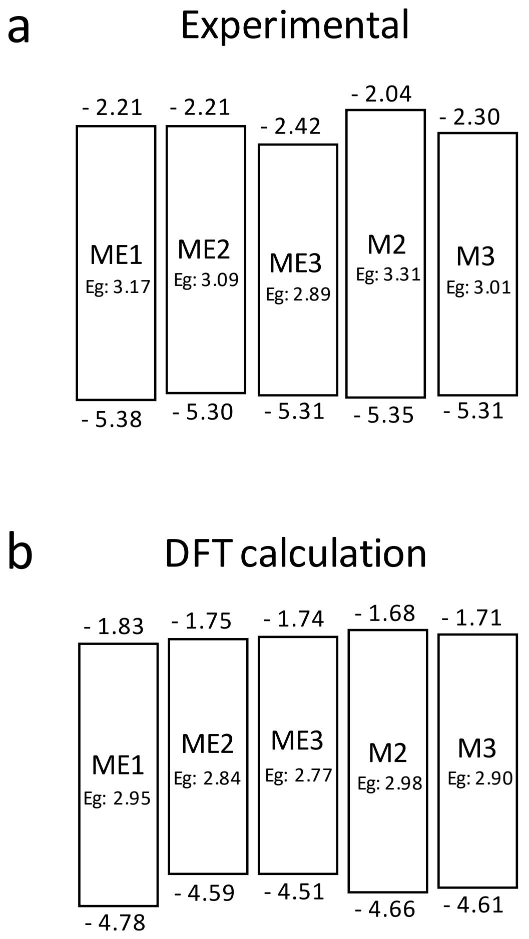

Supplementary Figure 20 | Energy levels of molecules calculated by **a**, cyclic voltammetry (CV) and **b**, density functional theory (DFT).

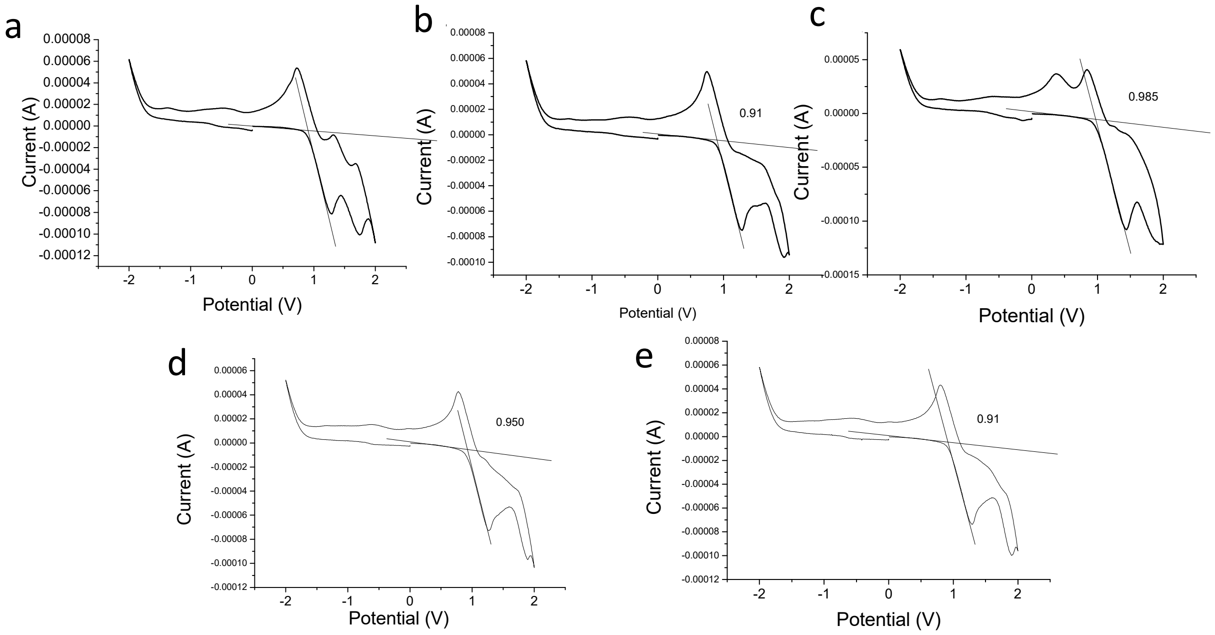

Supplementary Figure 21 | Cyclic voltammetry (CV) of **a**, ME2, **b**, ME3, **c**, ME1, **d**, M2, and **e**, M3.

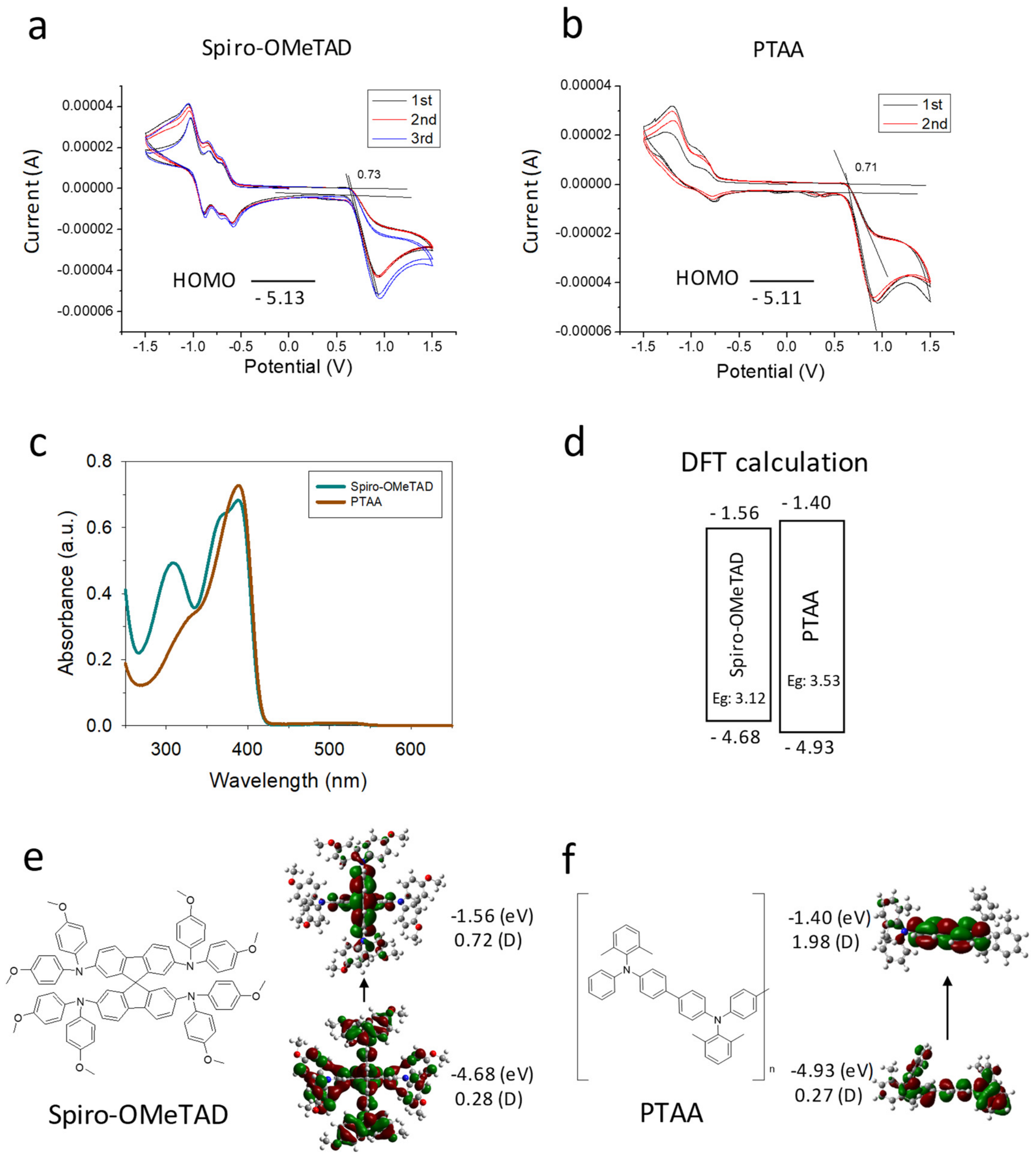

Supplementary Figure 22 | Cyclic voltammetry (CV) results of **a**, spiro-OMeTAD and **b**, PTAA. **c**, Absorbance of spiro-OMeTAD and PTAA. Experimentally estimated energy levels are added to (a) and (b). **d**, Theoretically calculated energy levels using DFT. Chemical structures and orbital diagrams calculated by DFT: **e**, spiro-OMeTAD and **f**, PTAA.

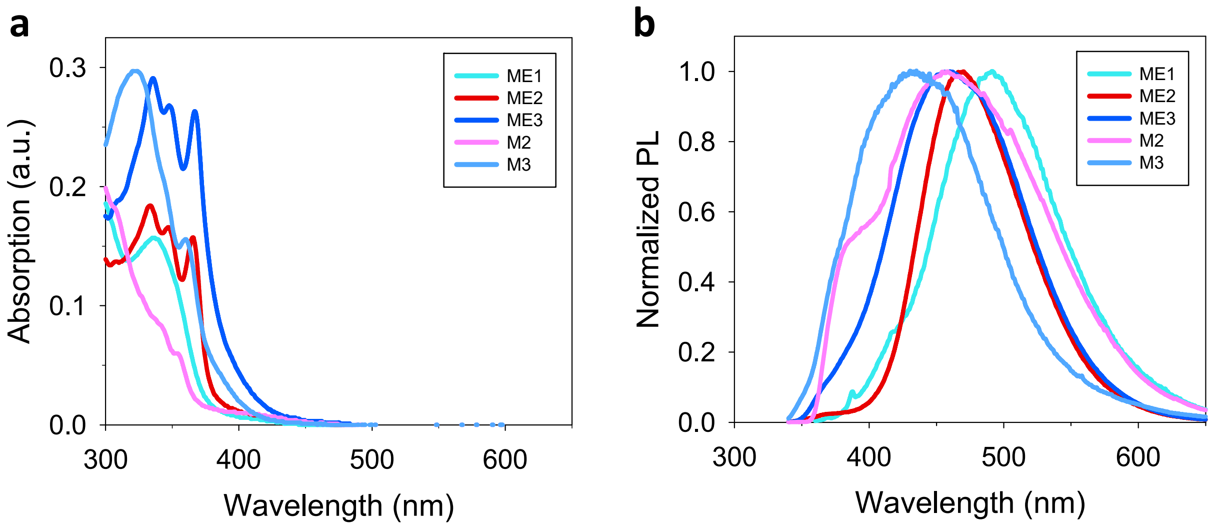

Supplementary Figure 23 | Photophysical properties of organic HTMs. **a**, Absorbance and **b**, steady-state photoluminescence of designed organic molecules in solution (1×10^{-5} M in $CHCl_3$) at room temperature.

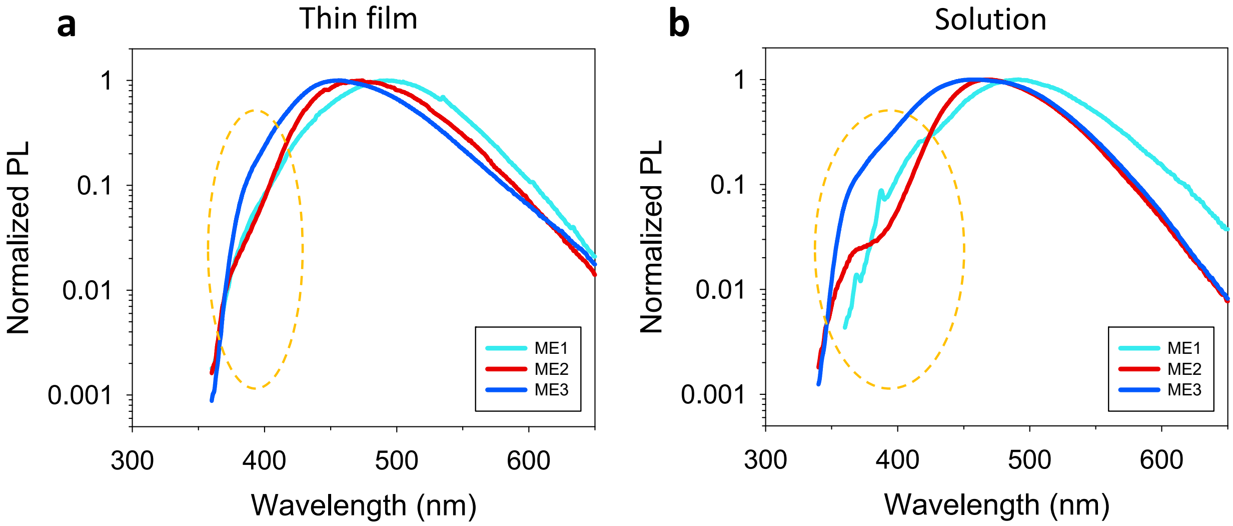

Supplementary Figure 24 | Steady-state photoluminescence signals of ESIPT-active molecules (log-scaled y-axis). **a**, In film (casted on quartz). **b**, In solution (1×10^{-5} M in $CHCl_3$) at room temperature.

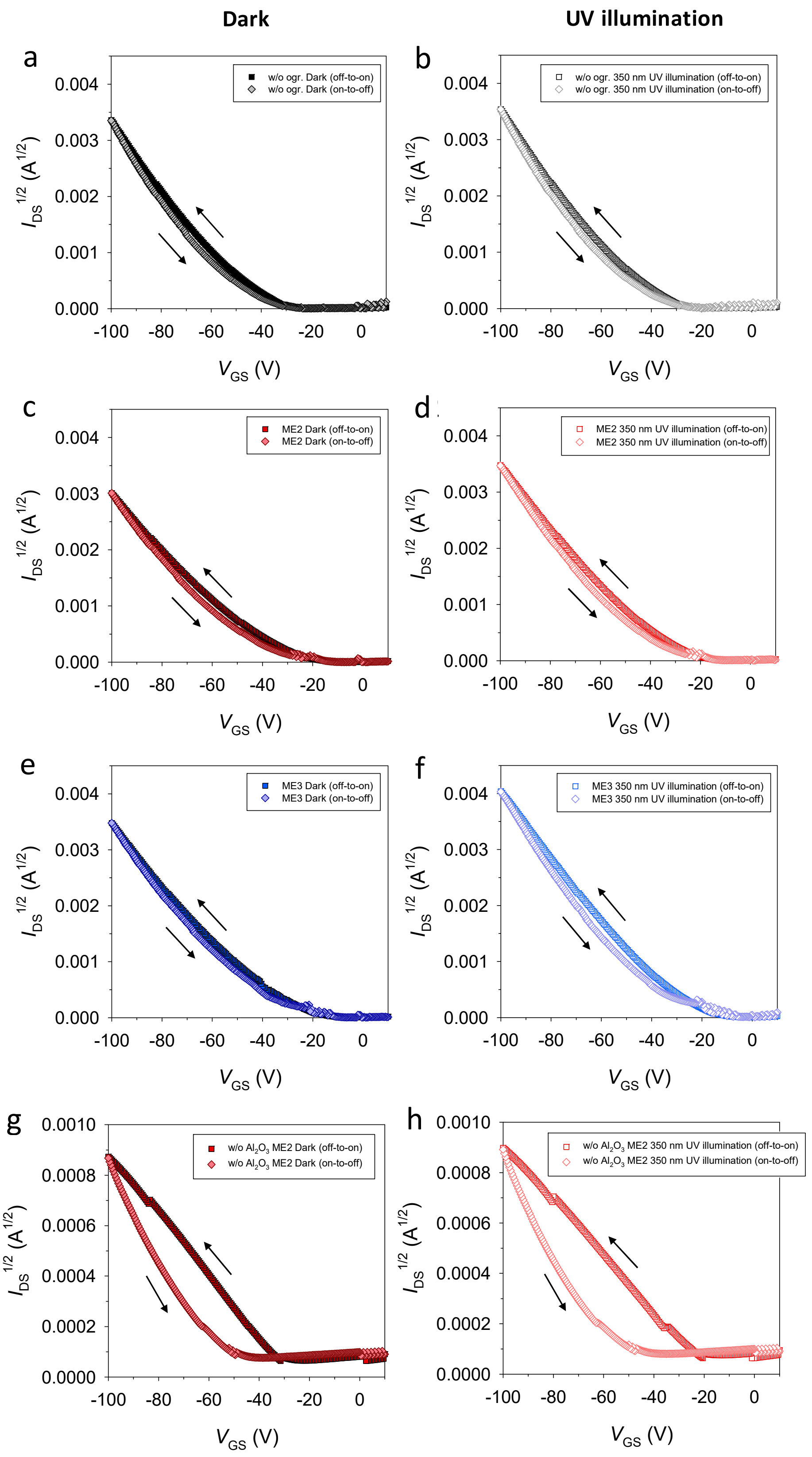

Supplementary Figure 25 | Transfer characteristics (I_{DS} vs. V_{GS}) of OFETs utilizing organic HTMs as gate dielectric with and without UV illumination (V_{DS} = -80 V), scanned in different directions. **a-f**, OFETs fabricated with Al_2O_3: (a, b) No organic HTM, (c, d) ME2, (e, f) ME3. **g-h**, OFET fabricated without Al_2O_3 (ME2 is utilized as gate dielectric). (a, c, e, g) Measured in dark. (b, d, f, h) Measured with 350 nm UV illumination.

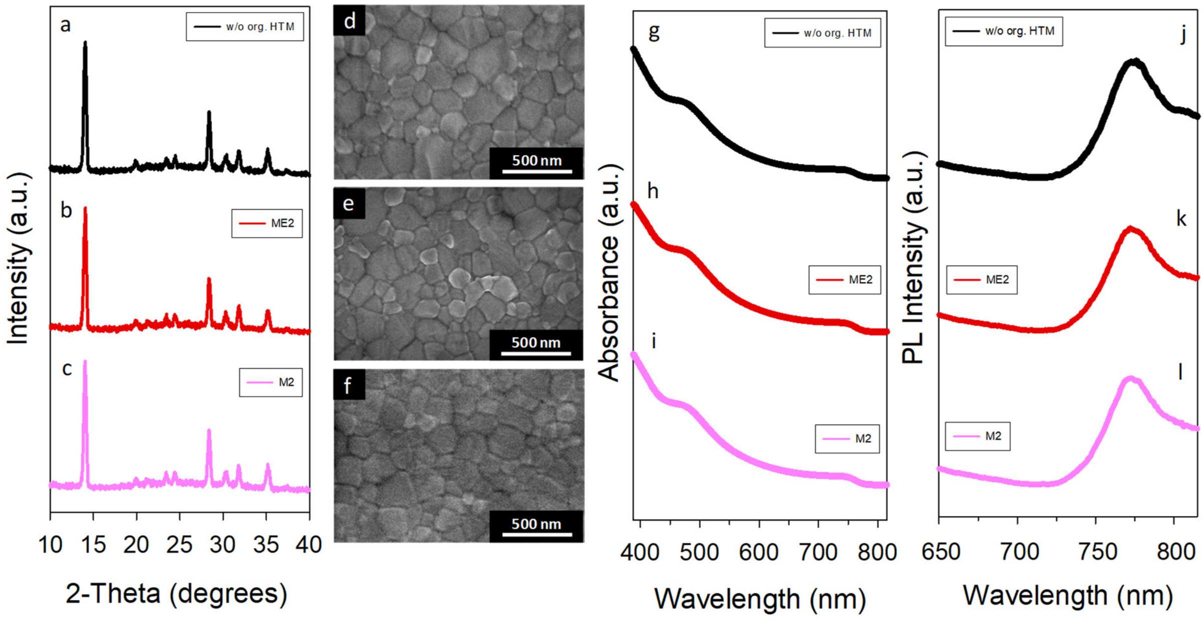

Supplementary Figure 26 | Characteristics of perovskite layers with and without organic HTMs. **a**, **b**, **c**, X-ray diffraction (XRD). **d**, **e**, **f**, Scanning electron microscopy (SEM). **g**, **h**, **i**, Absorbance. **j**, **k**, **l**, Photoluminescence (PL): (a, d, g, j) without organic HTM. (b, e, h, k) ME2. (c, f, i, l) M2.

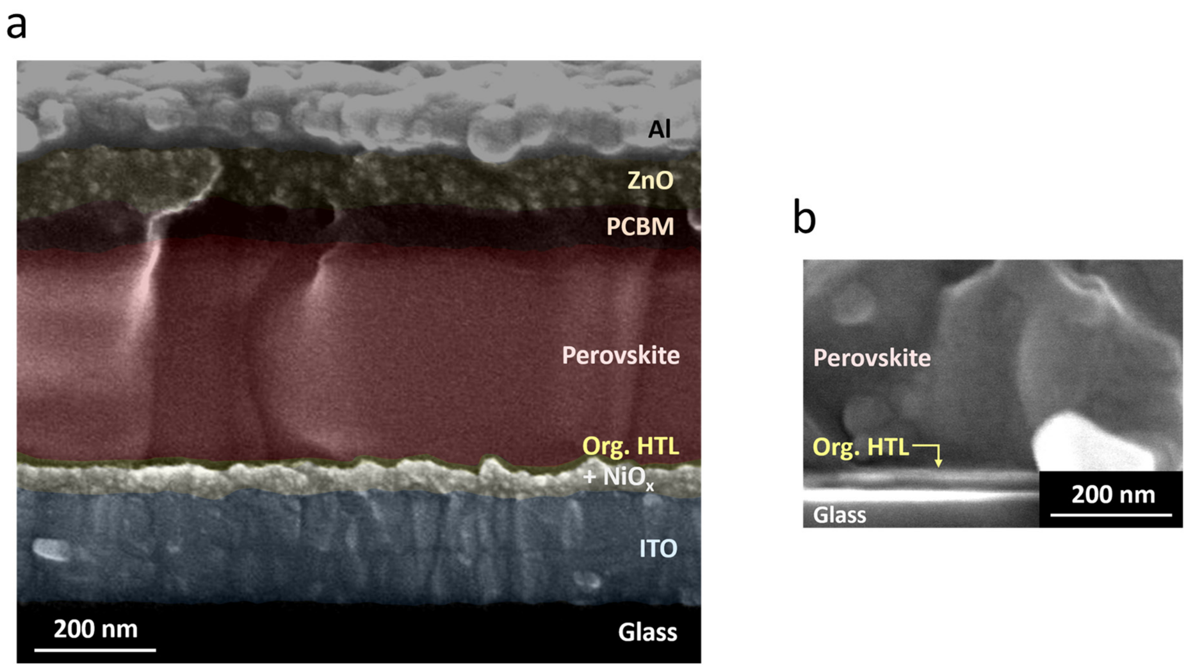

Supplementary Figure 27 | a, Cross-sectional SEM image of PSC device (glass/ITO/ NiO_x/organic HTM/perovskite/PCBM/ZnO/Al, ME2 applied). **b**, Cross-sectional SEM image of the sample having glass/organic HTM/perovskite stack (ME2 applied) utilized for controlling the thickness of organic HTM. The same process condition was utilized to form the organic HTM (ME2) layers of both (a) and (b) structures.

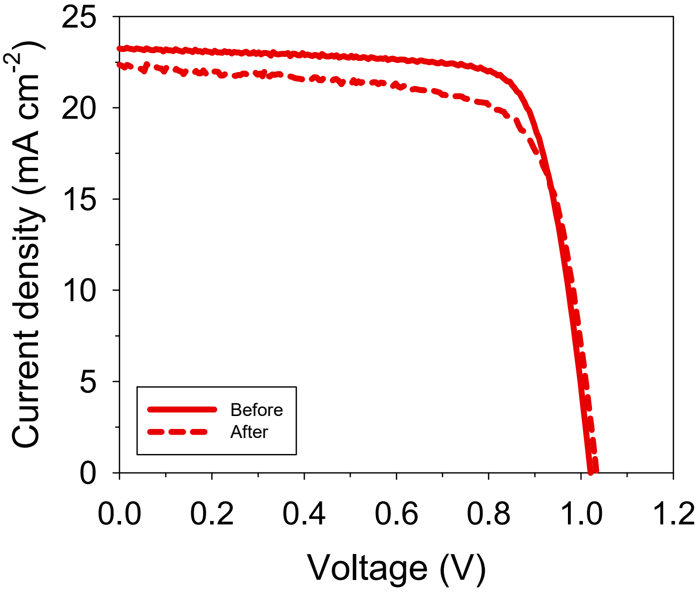

Supplementary Figure 28 | *J-V* characteristics of one of the devices used for KPFM measurement (ME2-applied) before (pristine device: J_{sc} = 23.25 mA cm^2, V_{oc} = 1.02 V, FF = 0.76, PCE = 18.13 %) and after (cleaved device: J_{sc} = 22.35 mA cm^2, V_{oc} = 1.03 V, FF = 0.72, PCE = 16.65 %) measurement.

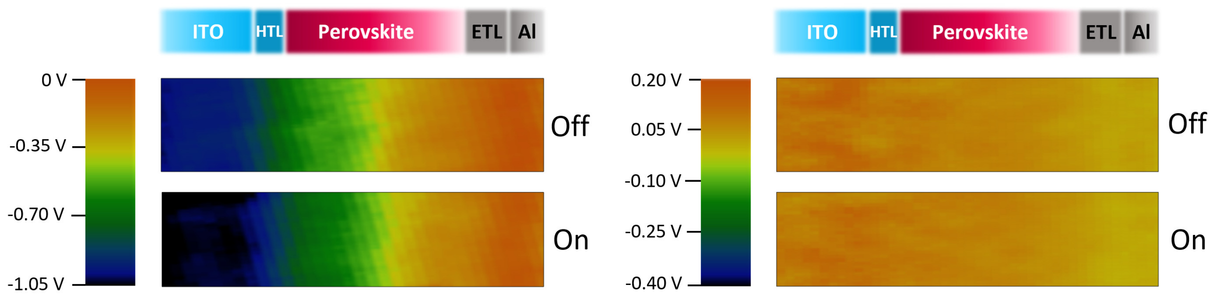

Supplementary Figure 29 | CPD maps of KPFM measurement (ME2-applied PSC). **a**, Reverse bias condition (around -0.5 V). **b**, Forward bias condition (around 0.8 V).

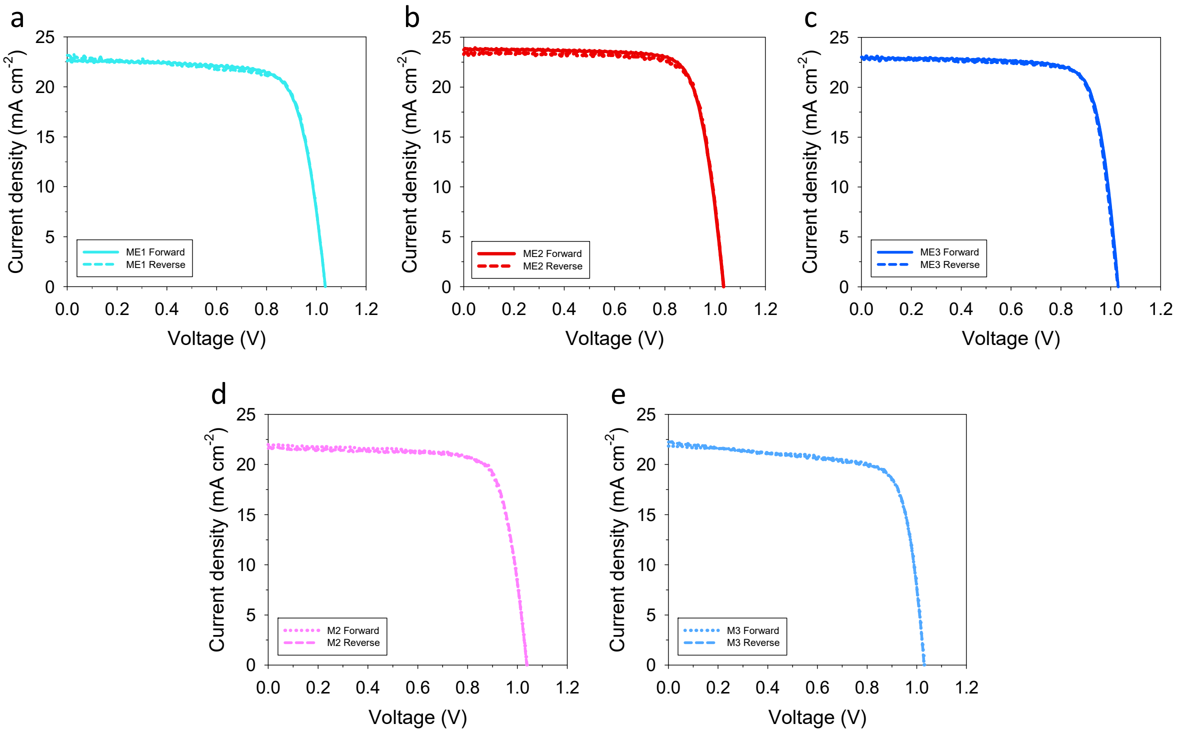

Supplementary Figure 30 | *J*–*V* curves of **a**, ME1-, **b**, ME2-, **c**, ME3-, **d**, M2- and **e**, M3-applied PSCs, scanned in forward and reverse directions. All data were measured at AM 1.5 G with an intensity of 100 mW cm^{-2}.

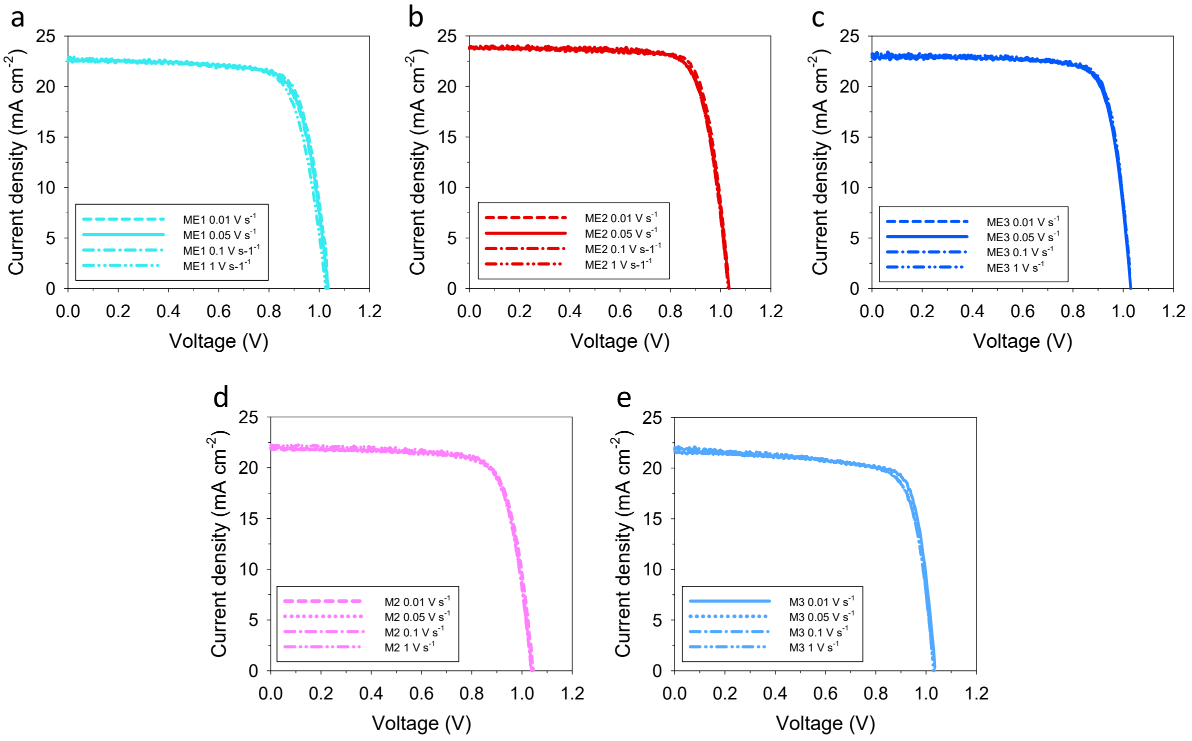

Supplementary Figure 31 | *J–V* curves of **a**, ME1-, **b**, ME2-, **c**, ME3-, **d**, M2- and **e**, M3-applied PSCs, according to the scan rate in forward direction. All data were measured at AM 1.5 G with an intensity of 100 mW cm^{-2}.

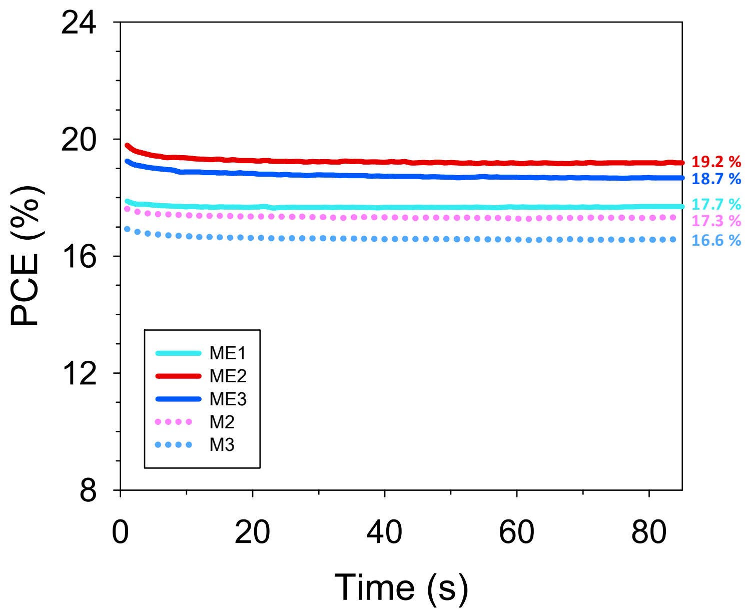

Supplementary Figure 32 | PCE measured at the maximum power point (MPP) for a ME1-, ME2-, ME3-, M2- and M3-applied PSCs.

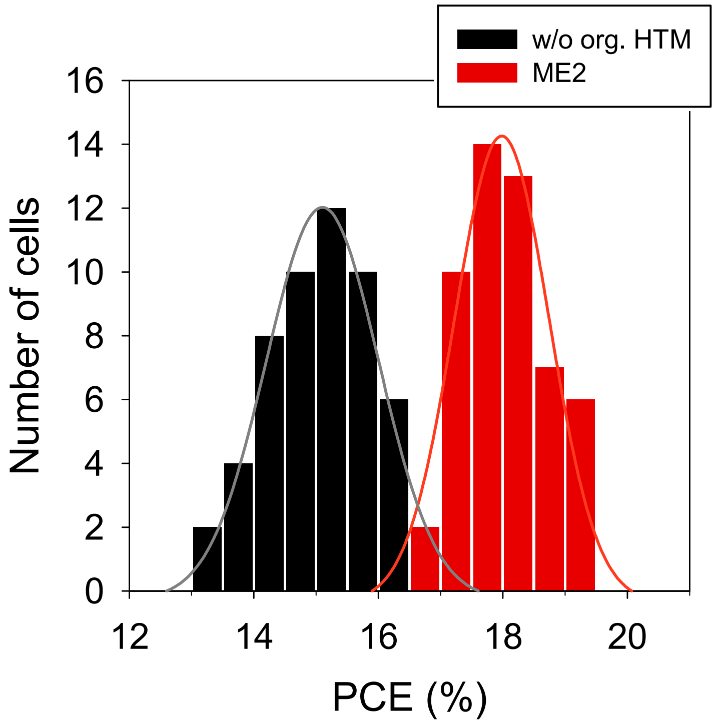

Supplementary Figure 33 | Histograms of power conversion efficiency (PCE) of PSCs (black color: ITO/NiO_x/perovskite/PCBM/ZnO/Al and red color: ITO/NiO_x/ME2/perovskite/PCBM/ZnO/Al). 52 cells for each case.

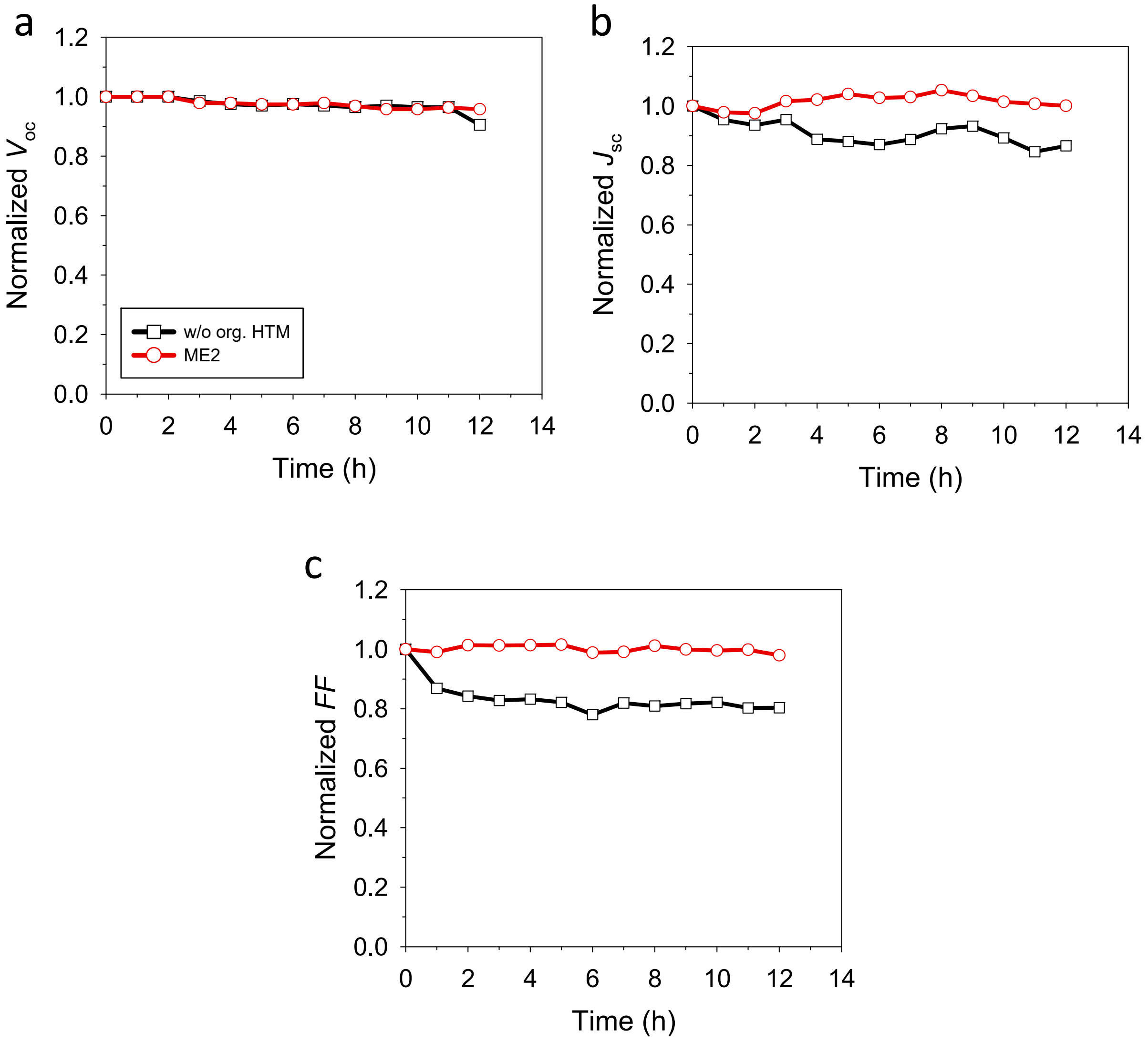

Supplementary Figure 34 | Photostability tests under constant AM 1.5G illumination with a xenon lamp, including UV radiation, without encapsulation in air (black line: ITO/NiO_x/perovskite/PCBM/ZnO/Al and red line: ITO/NiO_x/ME2/perovskite/PCBM/ZnO/Al). **a**, Open-circuit voltage (V_{oc}). **b**, Short-circuit current density (J_{sc}). **c**, Fill factor (FF).

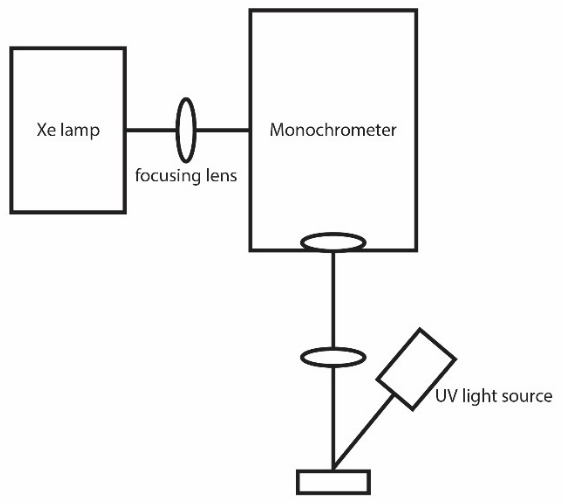

Supplementary Figure 35 | Modified IPCE set-up (UV-assisted EQE measurement).

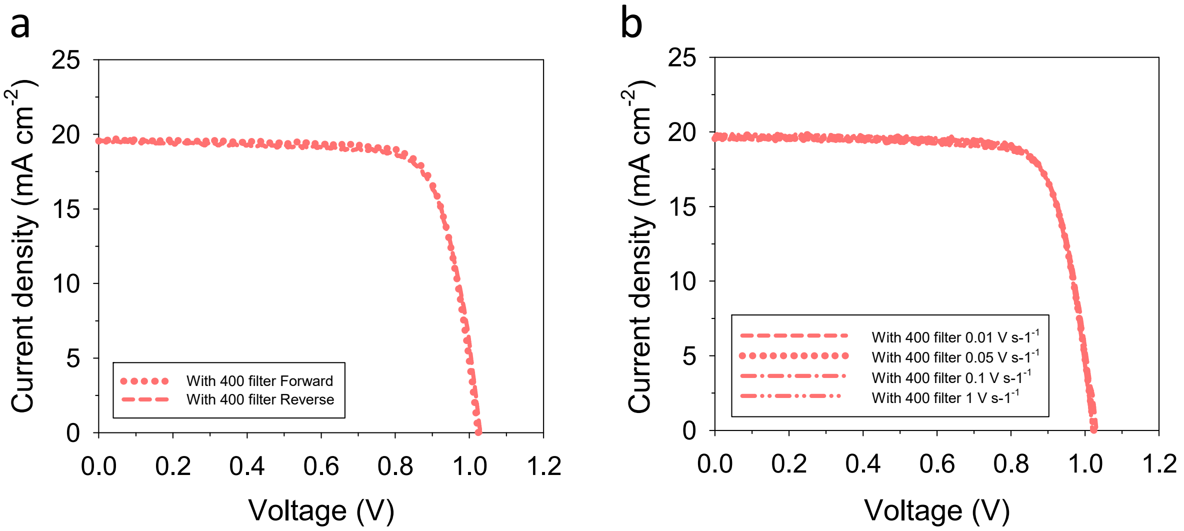

Supplementary Figure 36 | *J–V* curves of a ME2-applied PSCs, according to **a**, scan direction and **b**, scan rate with 400 nm filter. All data were measured at AM 1.5 G with an intensity of 100 mW cm^{-2}.

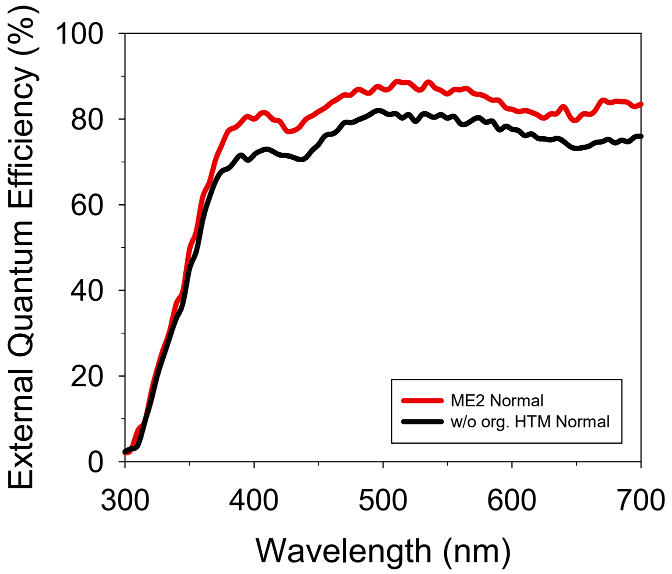

Supplementary Figure 37 | The comparison of EQE of the device without organic HTM and that with ME2 in UV region, measured by the conventional IPCE set-up.

Supplementary Table 1 | Fitted parameters of PL decay curves of organic HTMs on quartz samples (the values of the goodness-of-fit parameter (χ^2) are all close to 1.0).

	A_1	τ_1(ns)	A_2	τ_2(ns)	A_3	τ_3(ns)	τ_{avg}(ns)
ME2	59 %	0.5	32 %	2.8	9 %	10.7	2.1
M2	94 %	0.1	4 %	1.1	2 %	6.5	0.2

where, $Counts(t) = A_1\exp(-t/\tau_1) + A_2\exp(-t/\tau_2) + A_3\exp(-t/\tau_3)$

τ_{avg}: amplitude weighted average lifetime

PL decays of spiro-OMeTAD and PTAA are too fast to fit their curves.

Supplementary Table 2 | Device performances of PSCs with various organic HTMs (over 50 devices for each condition).

HTM	V_{oc} (V)	J_{sc} (mA cm^{-2})	FF	PCE (%)
w/o org. HTM	1.00 ± 0.03 (1.04)	20.37 ± 1.23 (20.72)	0.74 ± 0.03 (0.76)	14.97 ± 0.83 (16.31)
ME1	1.00 ± 0.02 (1.04)	22.13 ± 1.49 (22.58)	0.74 ± 0.03 (0.76)	16.37 ± 1.03 (17.85)
ME2	1.00 ± 0.02 (1.03)	23.55 ± 0.82 (23.74)	0.77 ± 0.02 (0.78)	18.03 ± 0.65 (19.22)
ME3	1.00 ± 0.02 (1.03)	22.71 ± 0.98 (23.02)	0.76 ± 0.03 (0.79)	17.26 ± 1.21 (18.73)
M2	1.00 ± 0.03 (1.04)	21.55 ± 1.10 (21.98)	0.74 ± 0.03 (0.76)	15.95 ± 0.78 (17.37)
M3	1.00 ± 0.02 (1.03)	21.36 ± 1.30 (21.84)	0.74 ± 0.03 (0.75)	15.81 ± 1.06 (16.87)
Spiro-MeOTAD	0.98 ± 0.02 (1.01)	20.48 ± 1.36 (21.12)	0.75 ± 0.04 (0.76)	15.13 ± 1.27 (16.36)
PTAA	0.96 ± 0.02 (0.99)	20.63 ± 1.18 (21.27)	0.70 ± 0.03 (0.72)	14.01 ± 1.11 (15.16)

Values in parentheses are from the best performing device.
± values are standard deviation.

Supplementary Table 3 | Fitted parameters of PL decay curves of perovskite on organic HTMs, excited by 375 nm laser (the values of the goodness-of-fit parameter (χ^2) are all close to 1.0).

	A_1	τ_1(ns)	A_2	τ_2(ns)	A_3	τ_3(ns)	τ_{avg}(ns)
NiO_x/ME2/perovskite	16 %	0.3	56 %	2.3	28 %	6.9	3.3
NiO_x/M2/perovskite	66 %	2.4	33 %	10.4	2 %	38.8	5.6
NiO_x/perovskite	29 %	1.1	42 %	6.3	29 %	15.7	7.6
Perovskite	45 %	5.1	51 %	17.9	4 %	50.6	13.5

where, $Counts(t) = A_1\exp(-t/\tau_1) + A_2\exp(-t/\tau_2) + A_3\exp(-t/\tau_3)$
τ_{avg}: amplitude weighted average lifetime

Supplementary Table 4 | Fitted parameters of PL decay curves of perovskite on organic HTMs, excited by 670 nm laser (the values of the goodness-of-fit parameter (χ^2) are all close to 1.0).

	A_1	τ_1(ns)	A_2	τ_2(ns)	A_3	τ_3(ns)	τ_{avg}(ns)
NiO_x/ME2/perovskite	52 %	2.5	45 %	9.3	3 %	52.1	7.1
NiO_x/M2/perovskite	47 %	2.8	49 %	9.7	4 %	51.1	8.0
NiO_x/perovskite	42 %	2.6	54 %	9.6	4 %	51.8	8.1
Perovskite	33 %	4.4	62 %	15.7	5 %	58.9	14.0

where, $Counts(t) = A_1\exp(-t/\tau_1) + A_2\exp(-t/\tau_2) + A_3\exp(-t/\tau_3)$
τ_{avg}: amplitude weighted average lifetime

Supplemetary Note 1 | Detailed ^{1}H NMR, ^{13}C NMR and MS data of 2-(1-(4-(10H-phenoxazin-10-yl)phenyl)-4,5-diphenyl-1H-imidazol-2-yl)-4-methoxyphenol (ME1)

^{1}H NMR ($CDCl_3$) δ 12.79 (s, 1H), 7.60 (d, J = 7.2 Hz, 2H), 7.44 (d, J = 8.2 Hz, 2H), 7.37 (dd, J = 12.4, 4.0 Hz, 2H), 7.33 – 7.27 (m, 5H), 7.20 (d, J = 6.9 Hz, 2H), 7.05 (d, J = 8.9 Hz, 1H), 6.82 (dd, J = 8.9, 2.9 Hz, 1H), 6.69 (dt, J = 14.7, 7.4 Hz, 5H), 6.64 – 6.56 (m, 2H), 6.39 (d, J = 2.9 Hz, 1H), 5.77 (d, J = 7.8 Hz, 2H), 3.49 (d, J = 2.9 Hz, 3H). ^{13}C NMR ($CDCl_3$) δ 152.55 (s), 151.24 (s), 144.62 (s), 143.91 (s), 139.86 (s), 137.31 (s), 133.67 (s), 132.82 (s), 132.42 (s), 131.73 (s), 131.43 (s), 130.52 (s), 129.77 (s), 128.62 (s), 128.52 (s), 128.35 (s), 127.21 (s), 126.91 (s), 123.16 (s), 121.85 (s), 118.37 (s), 116.55 (s), 115.72 (s), 112.99 (s), 112.58 (s), 111.28 (s), 55.47 (s). MS (EI) calcd. For $C_{40}H_{29}N_3O_3$: 599.22, found: 599. Anal. Calcd for $C_{40}H_{29}N_3O_3$: C, 80.11; H, 4.87; N, 7.01; O, 8.00 found: C, 80.31; H, 4.80; N, 7.02; O, 7.87.

Supplemetary Note 2 | Detailed ^{1}H NMR, ^{13}C NMR and MS data of 4-(10H-phenoxazin-10-yl)-2-(1-phenyl-1H-phenanthro[9,10-d]imidazol-2-yl)phenol (ME2)

^{1}H NMR ($CDCl_3$) *δ* 13.63 (s, 1H), 8.80 – 8.68 (m, 3H), 7.79 (t, *J* = 7.5 Hz, 1H), 7.74 – 7.66 (m, 1H), 7.57 – 7.44 (m, 6H), 7.29 (t, *J* = 7.4 Hz, 1H), 7.24 (s, 1H), 7.19 – 7.11 (m, 2H), 6.62 (dd, *J* = 6.5, 3.5 Hz, 5H), 6.58 – 6.48 (m, 2H), 5.73 (d, *J* = 8.2 Hz, 2H). ^{13}C NMR ($CDCl_3$) δ 158.54 (s), 147.43 (s), 143.78 (s), 138.26 (s), 134.23 (s), 133.05 (s), 130.96 (s), 130.61 (s), 129.59 (s), 129.28 (s), 128.54 (s), 127.61 (s), 127.25 – 127.13 (m), 126.63 (s), 126.27 (s), 125.56 (s), 124.18 (s), 123.25 (s), 123.12 (s), 122.61 (s), 122.44 (s), 121.01 (d, *J* = 7.2 Hz), 120.29 (s), 115.11 (s), 114.96 (s), 113.50 (s). MS (EI) calcd. For $C_{39}H_{25}N_3O_2$: 567.19, found: 567. Anal. Calcd for $C_{39}H_{25}N_3O_2$: C, 82.52; H, 4.44; N, 7.40; O,5.64 found: C, 82.68; H, 4.39; N, 7.01; O, 5.92.

Supplemetary Note 3 | Detailed ^{1}H NMR, ^{13}C NMR and MS data of 5-(10H-phenoxazin-10-yl)-2-(1-phenyl-1H-phenanthro[9,10-d]imidazol-2-yl)phenol (ME3)

^{1}H NMR ($CDCl_3$) δ 14.35 (s, 1H), 8.78 (d, J = 8.3 Hz, 1H), 8.72 (d, J = 8.0 Hz, 2H), 7.78 (d, J = 7.1 Hz, 4H), 7.69 (t, J = 8.7 Hz, 3H), 7.55 (t, J = 7.6 Hz, 1H), 7.29 (d, J = 7.8 Hz, 1H), 7.17 (s, 1H), 7.06 (d, J = 8.2 Hz, 1H), 6.93 (d, J = 8.5 Hz, 1H), 6.71 – 6.53 (m, 6H), 6.52 – 6.45 (m, 1H), 6.08 (d, J = 7.4 Hz, 2H). ^{13}C NMR ($CDCl_3$) δ 161.58 (s), 147.63 (s), 143.91 (s), 140.92 (s), 138.82 (s), 134.27 (s), 133.80 (s), 131.44 – 131.29 (m), 131.02 (d, J = 20.4 Hz), 129.64 (s), 128.98 (s), 128.54 (s), 128.10 (d, J = 11.7 Hz), 127.62 (s), 127.18 (s), 126.45 (d, J = 38.7 Hz), 126.19 – 126.11 (m), 125.58 (d, J = 9.9 Hz), 124.24 (s), 123.21 (s), 122.53 (d, J = 9.9 Hz), 122.09 – 120.83 (m), 120.83 – 120.42 (m), 120.12 (d, J = 13.6 Hz), 119.70 (s), 115.44 (s), 113.52 (d, J = 14.7 Hz), 113.20 (s). MS (EI) calcd. For $C_{39}H_{25}N_3O_2$: 567.19, found: 567. Anal. Calcd for $C_{39}H_{25}N_3O_2$: C, 82.52; H, 4.44; N, 7.40; O, 5.64 found: C, 82.44; H, 4.55; N, 7.36; O, 5.65.

Supplemetary Note 4 | Detailed ^{1}H NMR, ^{13}C NMR and MS data of 10-(4-methoxy-3-(1-phenyl-1H-phenanthro[9,10-d]imidazol-2-yl)phenyl)-10H-phenoxazine (M2)

^{1}H NMR ($CDCl_3$) δ 8.85 (d, J = 7.1 Hz, 1H), 8.77 (d, J = 8.1 Hz, 1H), 8.71 (d, J = 8.0 Hz, 1H), 7.76 – 7.68 (m, 1H), 7.67 – 7.61 (m, 1H), 7.57 – 7.37 (m, 7H), 7.33 (s, 1H), 7.28 (s, 1H), 7.26 – 7.23 (m, 1H), 7.00 (d, J = 8.4 Hz, 1H), 6.69 – 6.60 (m, 4H), 6.60 – 6.53 (m, 2H), 5.70 (d, J = 7.4 Hz, 2H), 3.76 (s, 3H). ^{13}C NMR ($CDCl_3$) δ 158.11 (s), 143.82 (s), 138.06 (s), 134.96 (s), 134.40 (s), 133.55 (s), 130.75 (s), 129.32 (d, J = 11.2 Hz), 128.51 (s), 128.17 (s), 127.26 (s), 126.23 (s), 125.49 (s), 124.96 (s), 124.06 (s), 123.14 (d, J = 11.7 Hz), 122.77 (s), 121.23 (s), 120.90 (s), 115.30 (s), 113.27 (s), 113.06 (s), 55.70 (s). MS (EI) calcd. For $C_{40}H_{27}N_3O_2$: 581.21, found: 581. Anal. Calcd for $C_{40}H_{27}N_3O_2$: C, 82.60; H, 4.68; N, 7.22; O, 5.50 found: C, 82.67; H, 4.71; N, 7.10; O, 5.52.

Supplemetary Note 5 | Detailed ^{1}H NMR, ^{13}C NMR and MS data of 10-(4-(1-phenyl-1H-phenanthro[9,10-d]imidazol-2-yl)phenyl)-10H-phenoxazine (M3)

^{1}H NMR ($CDCl_3$) δ 8.92 (d, J = 7.8 Hz, 1H), 8.79 (d, J = 8.4 Hz, 1H), 8.73 (d, J = 8.3 Hz, 1H), 7.84 – 7.80 (m, 2H), 7.77 (t, J = 7.5 Hz, 1H), 7.71 – 7.62 (m, 4H), 7.61 – 7.56 (m, 2H), 7.54 (t, J = 7.6 Hz, 1H), 7.28 (d, J = 8.3 Hz, 3H), 7.22 (d, J = 7.9 Hz, 1H), 6.70 (dd, J = 7.7, 1.7 Hz, 2H), 6.67 (dd, J = 7.2, 1.2 Hz, 2H), 6.64 – 6.61 (m, 1H), 6.59 (dd, J = 7.4, 1.7 Hz, 1H), 5.94 – 5.90 (m, 2H). ^{13}C NMR ($CDCl_3$) δ 149.75 (s), 143.91 (s), 139.41 (s), 138.59 (s), 137.49 (s), 134.02 (s), 131.89 (s), 130.71 (s), 130.29 (s), 130.09 (s), 129.41 (s), 129.06 (s), 128.35 (s), 128.23 (s), 127.38 (s), 127.13 (s), 126.36 (s), 125.79 (s), 125.11 (s), 124.16 (s), 123.18 (d, J = 3.3 Hz), 122.95 (s), 122.74 (s), 121.48 (s), 120.88 (s), 115.50 (s), 113.28 (s). MS (EI) calcd. For $C_{39}H_{25}N_3O$: 551.20, found: 551. Anal. Calcd for $C_{39}H_{25}N_3O$: C, 84.91; H, 4.57; N, 7.62; O, 2.90 found: C, 84.60; H, 4.69; N, 7.67, O, 3.04. We noticed that M3 was reported in *J. Nanosci. Nanotechnol.* 16, 11014-11017 (2016) after the submission of manuscript. However, this molecule is not a key molecule having both high transition dipole and extended lifetime at excitation that we claimed in our manuscript, and it is utilized as non-proton-transfer model compounds as a comparison to the ESIPT-enabled ME3 in our work. In that article, it was not also studied as HTM for solar cell. We believe that this would not affect the main claims in our manuscript.
